# Analysis of factors influencing the degree of accidental injury of bicycle riders considering data heterogeneity and imbalance

**DOI:** 10.1371/journal.pone.0301293

**Published:** 2024-05-14

**Authors:** Xinchi Dong, Daowen Zhang, Chaojian Wang, Tianshu Zhang

**Affiliations:** 1 School of Automobile and Transportation, Xihua University, Chengdu, China; 2 Vehicle Measurement Control and Safety Key Laboratory of Sichuan Province, Xihua University, Chengdu, China; 3 Faculty of Engineering and Technology, Sichuan Sanhe College of Professionals, Luzhou, China; 4 Computer and Mathematical Sciences, The University of Adelaide, North Terrace Adelaide, Adelaide, Australia; Tsinghua University, CHINA

## Abstract

Bicycle safety has emerged as a pressing concern within the vulnerable transportation community. Numerous studies have been conducted to identify the significant factors that contribute to the severity of cyclist injuries, yet the findings have been subject to uncertainty due to unobserved heterogeneity and class imbalance. This research aims to address these issues by developing a model to examine the impact of key factors on cyclist injury severity, accounting for data heterogeneity and imbalance. To incorporate unobserved heterogeneity, a total of 3,895 bicycle accidents were categorized into three homogeneous sub-accident clusters using Latent Class Cluster Analysis (LCA). Additionally, five over-sampling techniques were employed to mitigate the effects of data imbalance in each accident cluster category. Subsequently, Bayesian Network (BN) structure learning algorithms were utilized to construct 32 BN models after pairing the accident data from the four accident cluster types before and after sampling. The optimal BN models for each accident cluster type provided insights into the key factors associated with cyclist injury severity. The results indicate that the key factors influencing serious cyclist injuries vary heterogeneously across different accident clusters. Female cyclists, adverse weather conditions such as rain and snow, and off-peak periods were identified as key factors in several subclasses of accident clusters. Conversely, factors such as the week of the accident, characteristics of the trafficway, the season, drivers failing to yield to the right-of-way, distracted cyclists, and years of driving experience were found to be key factors in only one subcluster of accident clusters. Additionally, factors such as the time of the crash, gender of the cyclist, and weather conditions exhibit varying levels of heterogeneity across different accident clusters, and in some cases, exhibit opposing effects.

## 1 Introduction

Bicycle travel is considered a sustainable and low-carbon mode of transportation with numerous socio-economic and personal health benefits [1]. However, cyclists are particularly vulnerable road users, lacking the protection afforded by motorists, and therefore, they are at a higher risk of sustaining fatal injuries in collisions [2]. Ensuring the safety of cyclists has become a major concern for traffic engineers and policymakers [3]. According to the World Health Organization (WHO), over 1.35 million people worldwide will lose their lives in road traffic crashes during 2020, with approximately 41,000 of these fatalities involving cyclists, accounting for 3% of global road traffic deaths [4]. Moreover, data on traffic fatalities from the National Highway Traffic Safety Administration (NHTSA) in 2018 revealed an alarming increase in cyclist deaths, reaching a nearly two-decade high [5]. With the rise of sustainable urban transportation and the growing popularity of bike-sharing programs, the use of bicycles on urban roads has surged, making bicycle safety a critically important issue for cyclists [6]. Therefore, it is imperative to investigate the factors that contribute to the severity of injuries in road bicycle accidents and develop targeted measures to mitigate the occurrence and reduce the severity of these incidents.

In general, researchers base their work on traffic accident data and use statistical methods or machine learning methods to identify the key factors that influence accident severity. In particular, traffic accidents are complex multifactorial events that occur under different conditions with a complex occurrence process, and the pattern of factor effects on accidents is not fully revealed, and significant unobserved heterogeneity can also exist. Because accidents are episodic events with a much lower number of serious and fatal categories than minor injuries, accident data usually suffer from category imbalance [7]. It is worth noting that if the problem of data heterogeneity and imbalance is ignored when constructing an analytical model of factors, it may lead to incorrect parameter estimation of the models, reduce the ability of the models to identify risk factors, or even draw biased inferences for safety analysis [8]. In addition, most of the existing research methods are based on derivative classes of parametric models with specific assumptions such as Logit and Probit, and the non-collinearity requirement of independent variables in this class of methods can limit the introduction of logically relevant variables, failing to fully explore risk factors [9].

Therefore, the present study aimed to reduce the impact of heterogeneity and imbalance among accident data on the analysis results as well as to achieve a quantitative analysis of accident factors. The rest of the paper is organized as follows: section 2 provides a literature review that summarizes previous studies in terms of content and methodology; Section 3 explains the study data; Section 4 describes the methodology used; Sections 5 and 6 provide an analysis and discussion of the results of the model calculations, respectively; and, finally, the main findings of this study are summarized in section 7.

## 2 Literature review

At present, scholars have explored various factors influencing the severity of cyclist injuries based on traffic accident data, which can be broadly classified into two categories, namely, micro factors and macro factors. Micro factors include cyclist factors and driver factors (age and gender), whereas macro factors include bicycle factors, vehicle factors (vehicle type and collision location), road factors (road speed limit and road conditions), environmental factors (weather and light conditions), and time factors (season and holidays) [10]. Fewer studies have performed macroscopic analysis, focusing on the numerical safety effects of socioeconomic, demographic, commuting, and infrastructure factors on injury severity or accident rates, which can provide a scientific basis for decision-makers to reduce cycling risks at the planning level.

In order to explore the relationship between different factors and traffic accidents, many scholars have applied regression analysis-type methods and confirmed that there are statistical associations between driver, vehicle, road, and environmental factors and traffic accident risk [11–13]. However, this methodology makes it difficult to identify specific influencing factors and their expressions and ignores the diversity of influencing factors and the unobservability of some factors [14]. In addition, when the regression analysis method is used to evaluate the frequency of traffic accidents, the discrete distribution of traffic accident data and the limited sample size, which are common in traffic accident data, may also affect the effectiveness of data fitting [15].

To this end, researchers have used machine learning models such as Decision Tree (DT) models, BN models, Neural Network (NN) models, and Support Vector Machines (SVM) for traffic accident rate prediction and analysis and accident risk evaluation, taking advantage of the strengths of machine learning models in dealing with small-sample, nonlinear, and high-dimensional traffic accident data [16–18]. Although these models have strong predictive and analytical capabilities, they are weak in explaining the differences between risk factors, ignoring the degree of influence of different risk factors on accident risk evaluation, and may also lead to biased estimation of traffic accident risk evaluation. Meanwhile, accident categories in accident data are unbalanced, and for dichotomous accidents, there are fewer fatal accidents than non-fatal accidents, which challenges the performance of machine learning models. Since machine learning algorithms inherently assume that the data categories are balanced, machine learning models built on unbalanced data are usually biased toward the majority of the categories in classification, resulting in samples from the minority of the categories being susceptible to misclassification. In addition, Ignoring the imbalanced nature of the data in the modeling process can also lead to incorrect estimation of the model parameters and failure to fully explore the relationship between factors and injury severity. There are two main types of solutions to the imbalance problem as follows: algorithm-level methods and data-level methods. The algorithm-level approach is mainly cost-sensitive learning, which defines a "cost matrix" to assign different misclassification costs to the desired category, but it is difficult to obtain the best misclassification costs for each category [19]. Therefore, scholars mostly use the data-level approach, i.e., data resampling. Domestic and foreign scholars have studied the influencing factors of accident categories using different resampling techniques combined with machine learning algorithms, such as Decision Trees (DT) and XG boost, and they have found that resampling effectively improves the classification performance of models in a few classes and the ability to identify risk factors.

Heterogeneity refers to the fact that the influence of certain factors incorporated into the model on the accident consequences is not constant in all cases, but may vary randomly, and it is one of the main problems of the modeling process. If the heterogeneity of the data is ignored in the modeling process, it can lead to incorrect estimation of model parameters or even to wrong inferences [20]. Because traditional statistical and machine learning methods cannot adequately observe the heterogeneity of data, models that can account for unobserved heterogeneity have been introduced into traffic accident analysis, in which random parameters Logit and LCA are widely used [21].

Although the heterogeneity of the data can be observed with the random parameter Logit model, the method presupposes that the random parameters obey a normal or uniform distribution and ignores the possibility that the random parameters follow a complex, multistate distribution. To overcome this drawback, the entire dataset can be divided into sub-datasets of different clusters, which maximizes both the heterogeneity effect of the inter-group dataset and the homogeneity of the intra-group dataset. Thus, potential category analysis based on the idea of clustering has been applied to the field of traffic safety analysis. Lin Z et al. [22] based on NHTSA bicycle crash data, the entire dataset was divided into seven sub-datasets using LCA, and models were constructed for the overall dataset and the seven sub-datasets. The sub-models had better-fit superiority than the individual models developed using the entire dataset. Some of the variables had significant effects only in specific groups, and the same variable may have different levels of effect in different groups.

Therefore, the present study proposed a two-stage method integrating LCA, over-sampling, and BN based on the Crash Report Sampling System (CRSS) database from 2016–2020, considering bicycle accidents as the research object. It is intended to reduce the influence of data heterogeneity and imbalance from the method level and realize the quantitative analysis of accident causal factors. From the application level, the focus of bicycle accident prevention is clarified, and then precise prevention and control strategies are implemented.

## 3 Data description and preprocessing

The National Highway Traffic Safety Administration (NHTSA) has collected motor vehicle traffic crash data since the early 1970s to support its mission to reduce motor vehicle traffic crashes, injuries, and deaths on our Nation’s trafficways. NHTSA uses data from many sources, including the Crash Report Sampling System (CRSS) [23]. CRSS is a sample of police-reported crashes involving all types of motor vehicles, pedestrians, and cyclists, ranging from property damage-only crashes to those that result in fatalities. To investigate the relationship between the severity of cyclist injuries and the influencing factors, the present study searched the CRSS database for bicycle accidents occurring in urban areas from 2016 to 2020. The inclusion criteria were as follows:

1. The 5-year crash data provide a larger sample size, while the shorter time span allows controlling for changes in road and traffic conditions nationwide and reducing the effect of temporal instability [24]; and
2. Because the sample size of BSV crashes was small and BMV crashes were the main type of fatal bicycle accidents, BMV crashes were mainly considered in the present study. In addition, only collisions involving a motor vehicle (motorcycles were excluded due to the small number) and a pedal bike were screened for one-to-one driver-cyclist correspondence to reduce the influence of other potential risk factors. The exclusion criteria were as follows: (a) variables not relevant to the present study; (b) when missing values were higher than 30%; and (c) when the number of crashes with a given value was more than 95% of the total number of crashes.

Data for 3895 bicycle accidents in urban areas were obtained, and the original database classified the accident severity into the following five classes:

No Apparent Injury is a situation where there is no reason to believe that the person received any bodily harm from the motor vehicle crash. There is no physical evidence of injury and the person does not report any change in normal function (1.41%).

Possible Injury is any injury reported or claimed that is not a fatal injury, suspected serious injury, or suspected minor injury. Examples include momentary loss of consciousness, possible concussion, claim of injury limping, complaint of pain, or nausea (38.64%).

Minor Injury is any injury that is evident at the scene of the crash, other than fatal or serious injuries. Examples include a lump on the head, abrasions, bruises, and minor lacerations (cuts on the skin surface with minimal bleeding and no exposure of deeper tissue/muscle) (47.88%).

Serious Injury is any injury other than fatal that results in one or more of the following (10.99%).

- Severe laceration resulting in exposure of underlying tissues/muscle/organs or resulting in significant loss of blood
- Broken or distorted extremity (arm or leg)
- Crush injuries
- Suspected skull, chest, or abdominal injury other than bruises or minor lacerations
- Significant burns (second and third-degree burns over 10 percent or more of the body)
- Unconsciousness when taken from the crash scene
- Paralysis

Fatal Injury is any injury that results in death within 30 days after the motor vehicle crash in which the injury occurred (1.08%).

Due to the small percentage of uninjured accidents and fatal accidents, the present study combined uninjured, possibly injured, and slightly injured as non-serious injuries, and the remaining accident categories were combined as serious injuries. Referring to related studies on the severity of cyclist injuries and combining the field characteristics of CRSS, 25 variables were selected from five aspects, namely, time characteristics, vehicle characteristics, road characteristics, environmental characteristics, and driver/cyclist characteristics. The final values of the variables and their coding are listed in Table 1.

**Table 1:** Variables and their values.

	Variables	Values				
		1	2	3	4	5
Crash characteristics	Season	Spring	Summer	Autumn	Winter	
	Day of Week	Working day	Weekend			
	Hour of Crash	Morning Peak (6:00–8:59)	Evening Peak (16:00–18:59)	Off-Peak		
Drivers characteristics	Gender	Male	Female			
	Age	18~25 years old	26~40 years old	41~65 years old	˃65 years old	
	Failure to yield	Yes	No			
	View was blocked	Yes	No			
	Inattention	Yes	No			
Cyclists characteristics	Gender	Male	Female			
	Age	˂18 years old	18~25 years old	26~40 years old	41~65 years old	˃65 years old
	Initial Direction of Travel	Forward	Reverse	Downward		
	Failure to Yield	Yes	No			
	Violation of Traffic Control Device	Yes	No			
	Inattention	Yes	No			
Vehicle characteristics	Pre-Event Movement	Going Straight	Turn left	Turn right	Start/Stop	Other
	Vehicle Type	Passenger Cars	Multi-purpose vehicle	Trucks / Buses		
	Manner of Collision	Front-to-Front	Left Sideswipe	Right Sideswipe		
	Age of vehicle	0~5 years	6~10 years	11~15 years	˃15 years	
Roadway characteristics	Roadway Surface Condition	Non-Trafficway or Driveway Access	Dry	Wet		
	Relation to Junction	Intersection	Near the intersection	Non-intersection		
	Cross Section	Travel Lanes	Bike Lanes/Paved Shoulders/Parking Lanes	Sidewalk/Cross Walk/Driveway Access		
Environmental characteristics	Weather	Clear	Cloudy	Rain/Snow and other bad weather		
	Light Condition	Daylight	Dark-Not Lighted	Dark-Lighted	Dawn/Dusk	
	Traffic Control Device	None	Traffic signals	Stop/Yield signs		

## 4 Methodology

The purpose of the present study was to model bicycle accident crash data and explore the influence of key factors of each type of accident cluster on the severity of cyclist injuries while considering data heterogeneity and imbalance. The following methods were used in the present study: (1) an LCA model was used to manage data heterogeneity; (2) ROS, SMOTE, and ADASYN methods were used to contend with data imbalance; and (3) the sampled accident data were paired with a BN model, and five evaluation indicators, such as Accuracy, G-mean, and area under the curve (AUC) values, were used to select the best BN model.

### 4.1 Latent Class Cluster Analysis (LCA)

LCA is a probabilistic model-based clustering analysis method that achieves its basic requirement of local independence by assuming the existence of a latent variable used to explain the association between explicit variables. LCA has several advantages over traditional cluster analysis as follows: (1) in LCA, the number of clusters does not need to be predetermined, and the optimal number of clusters can be determined based on various goodness-of-fit metrics [25]; and (2) LCA can handle various types of variables, including nominal, continuous, count, and categorical variables, without a standardization process.

There are explicit and latent variables in LCA. Explicit variables are variables that can be directly observed in life, i.e., fields included in the accident data (such as light conditions, cross-section of the traffic way, and crash locations). Latent variables are unobservable variables that are generally not included in the database. Suppose there are *C* potential classes in the crash dataset, and *γ*_*c*_ denotes the probability that an accident lies in the potential class cluster *c* (*c* = 1, 2, ⋯, *C*) with a sum of 1. Each accident *i* contains M attributes, i.e., M explicit variables, and *Z*_*im*_ denotes the level number of *m*-th explicit variable for the *i*-th accident, in which *Z*_*im*_ = 1,2,⋯,*r*_*m*_。 *ρ*_*m*_,*r*_*m*_|*c* denotes the probability that the number of levels of m-th variable in cluster c is *r*_*m*_, called the conditional probability. The total conditional probability of each response level of each explicit variable is 1. *R*_*m*_ is the total number of levels of m-th explicit variable, and *I*(*Z*_*im*_ = *r*_*m*_) is an indicator function when *Z*_*im*_ = *r*_*m*_ and *I* is equal to 1; otherwise, it equals 0. Then, the *i*-th incident under all potential categories of clusters has a certain possible response vector probability as follows:

$$P(Z=z)=\sum_{c=1}^{C}\gamma_{c}\prod_{m=1}^{M}\prod_{r_{m}=1}^{R_{m}}\rho_{m,\left.r_{m}\right|c}^{I\left(Z_{im}=r_{m}\right)}$$ (1)

In this paper, the coded independent variable data were stored in CSV format and imported into LatentGold 4.5 software for LCA. However, LCA does not know the most appropriate number of clusters from the beginning. The most appropriate number can be found by testing different models with different numbers of clusters. The allocation error of assigning crashes to potential classes can be minimized by choosing the optimal number of clusters [25]. This number can be determined by a number of methods for measuring allocation accuracy. We determined the optimal number of classifications based on fitting metrics, among which the commonly used metrics were Bayesian information criteria (BIC), Akaike information criterion (AIC), consistent Akaike information criterion (CAIC), and entropy [26]. The number of clusters with the smallest scores for AIC, BIC, and CAIC is considered to be the most appropriate number of clusters. In addition, increasing the number of clusters may not always result in a minimum information criterion. Therefore, calculating the percentage reduction in BIC values between different models is preferred [27]. An entropy measure between 0 and 1 indicates the quality of the clustering solution. It is essentially a weighted average of the posterior affiliation probabilities for each case. The closer the entropy value is to 1 indicates better clustering [25, 28].

### 4.2 ROS, SMOTE, and ADASYN sampling

Oversampling balances the minority samples with the majority samples by replicating or synthesizing the minority samples. Oversampling methods have the advantage of improving model performance without causing information loss, and the main oversampling methods include ROS, SMOTE, and ADASYN [29], which are used to manage the imbalance problem of self-accident data sets.

The basic principle of random oversampling is to randomly select and replicate a small number of class samples until they are comparable to the majority of class samples. This method solves the problem of information loss due to oversampling.

SMOTE, first proposed by Chawla et al. [30], is the most classical oversampling method, which combines the nearest neighbor algorithm and interpolation method to generate new minority class samples and alleviate the problem of model overfitting. The basic process of SMOTE is to calculate the Euclidean distance s between each minority class sample *x*_*i*_ and each remaining minority class sample to obtain the k nearest neighbor samples *x*_*ij*_ of *x*_*i*_. A new sample is generated for each nearest neighbor sample according to the following formula:

$$x_{new}=x_{i}+rand\left(0,\;1\right)*(x_{ij}-x_{i})$$ (2)

The basic concept of ADASYN is to assign different weights to the minority class samples according to their learning difficulty, in which the more difficult minority class samples will generate more synthetic data compared to the easier minority class samples.

### 4.3 BN

BN is a probabilistic graphical model consisting of nodes representing random variables and directed edges describing the dependencies of the random variables. BN, as a directed acyclic graph, defines the joint probability distribution of random variables with possible causal relationships, which can be described by the following formula:

$$BN=(N,E,P\left(X_{n}\right))$$ (3)

where *N* denotes the set of nodes; *E* denotes the set of directed edges between nodes; and *P*(*X*_*n*_) denotes the joint probability distribution of the set of nodes. The construction of BN models includes the following two parts: structure learning, which is the process of finding the optimal network structure by learning the observed data; and parameter learning, which is the process of quantifying the dependencies between variables. The current structure learning methods of BN can be divided into two major categories, namely, constraint-based methods and scoring search-based methods. To obtain a more effective BN model, a common algorithm of the two types of methods were used in the present study.

Among the constraint-based methods, the Three Phase Dependency Analysis (TPDA), which was proposed by Cheng J et al. [31] in 2002, was used, which can adapt not only to the case of known node ordering, but also to the case of unknown node ordering.

In the present study, the scoring-based search method had the following two parts: the search operator and the scoring function. The search operator uses the widely used and computationally efficient hill climbing (HC) algorithm, and the scoring function uses the BDeu scoring function proposed by Buntine [32] in 1991. After determining the BN structure, the robust expectation maximization (EM) algorithm was used for parameter estimation.

## 5 Results

### 5.1 Clustering results

**Fig 1:**
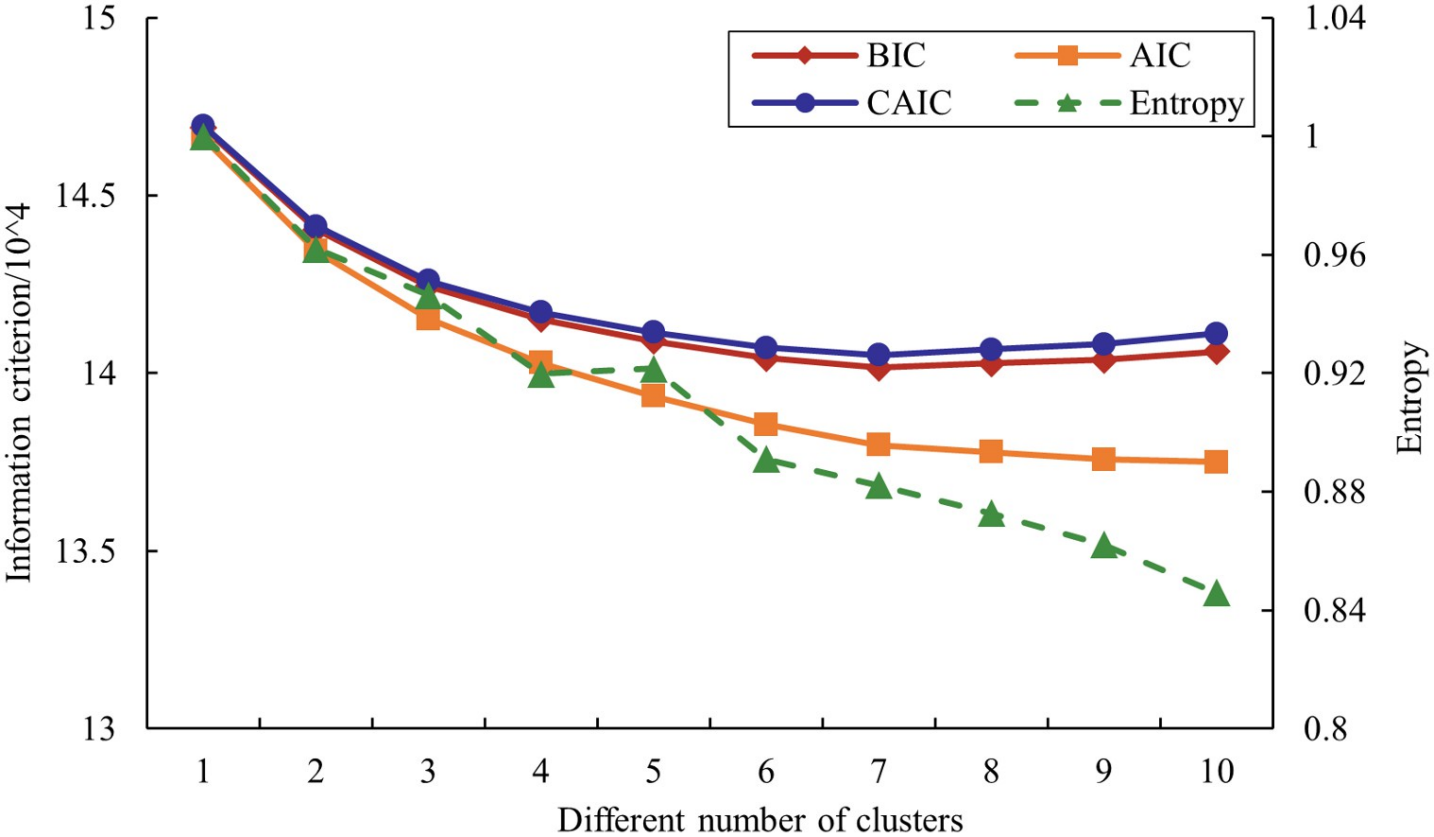
Information criterion and entropy value corresponding to different number of clusters.

The BIC, AIC, and CAIC values gradually decreased with the increase in cluster number (Fig 1). In general, lower IC values indicate better models. However, when analyzing large sample data, each IC value often does not reach the minimum value as the number of clusters increases. In this case, analyzing the rate of decline of each IC value is preferred. In addition, it has been argued that BIC is more reliable than AIC and CAIC when the amount of data is large. If the rate of decline of IC values is less than 1% and the entropy value is higher than 0.9, the number of clusters is considered to be the optimal number of clusters. In the present study, the decline rate of each IC value was lower than 1% in the M4 model, and the entropy value of the M3 model reached 0.9463, which is greater than 0.9, which indicates the clear separation between clusters and satisfactory fitness of the model. Considering the clustering effect of each model, the number of clusters in the present study was determined to be 3 [33].

LCA divided the overall bicycle accident data into three homogeneous traffic accident clusters, and the three accident clusters were defined as C1, C2, and C3 accident clusters. The category probabilities and conditional probabilities of the accident clusters are shown in Table 2.

**Table 2:** Distribution of featured variables describing cluster characteristics.

Variables	C1 (59.59)	C2 (29.46)	C3 (10.95)
Forward	48.67	70.13	25.20
Reverse	44.23	12.36	68.82
Downward	7.09	17.50	5.98
Going Straight	32.94	69.03	13.00
Turn left	21.75	12.09	10.68
Turn right	38.64	3.99	57.98
Start/Stop	5.82	2.24	12.74
Other	0.86	12.66	5.60
Non-Trafficway or Driveway Access	0.25	0.99	73.57
Dry	91.58	90.70	25.94
Wet	8.17	8.31	0.50
Intersection	91.93	20.80	3.72
Near the intersection	7.66	12.51	6.94
Non-intersection	0.41	66.69	89.34
Travel Lane	44.74	86.41	13.60
Lane/Paved Shoulder/Parking Lane	4.25	10.11	2.69
Sidewalk/Crosswalk/Driveway Access	51.02	3.48	83.71
None	13.87	92.78	81.19
Traffic signals	54.73	6.20	1.10
Stop/Yield signs	31.40	1.03	17.71

Note: the units of the figures in the table are "%".

After determining the number of clusters, it was necessary to identify the characteristic variables to determine the important characteristics of each type of accident cluster. Regarding accident cluster C1, 91.93% of the accidents occurred at intersections, 86.13% of the accidents had traffic control devices, 91.58% of the accidents were on dry roads, 38.64% of the vehicles were turning right at the time of the accident, 51.02% of the cyclists were on the Sidewalk/Crosswalk/Driveway Access, and 48.67% were the Forward. The main characteristics of the accidents were described as BMV accidents at intersections with traffic control devices. Similarly, the C2 accident cluster was described as non-intersection BMV accidents without traffic signal control devices, and the C3 accident cluster was described as BMV accidents on off-road/dedicated side streets without traffic control devices.

### 5.2 Results of the construction of the BMV BN model

**Fig 2:**
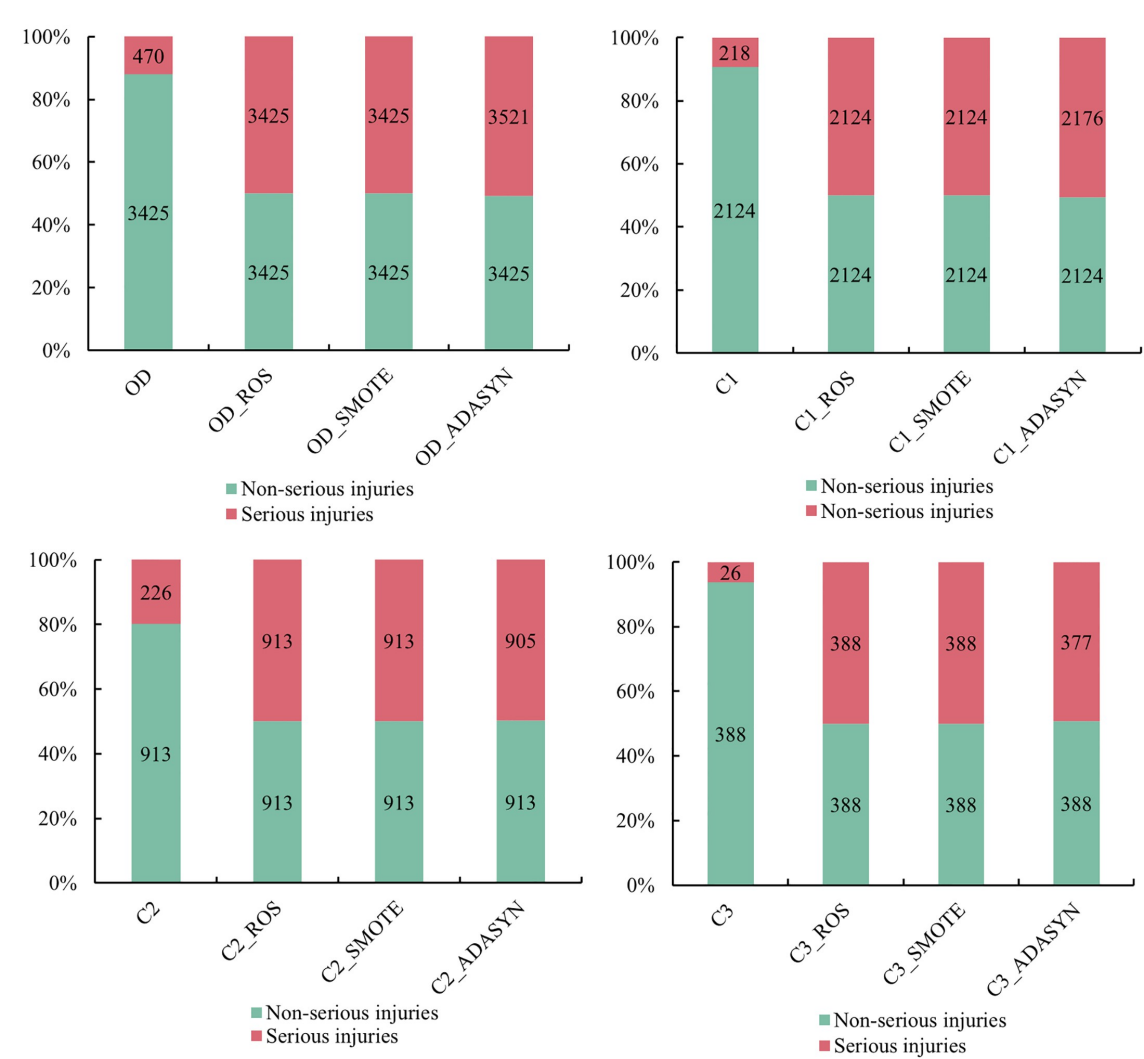
Distribution of accident categories before and after sampling.

The original accident clusters (OD accident clusters) as well as the C1, C2, and C3 accident clusters all had different degrees of category imbalance problems. To reduce the impact of accident data category imbalance, ROS, SMOTE, and ADASYN were used to resample various accident clusters. In Fig 2, OD, C1, C2, and C3 represent the corresponding accident clusters without balancing treatment; OD_X represents the balancing data obtained after sampling OD data using X. For example, OD_SMOTE indicates the balancing data obtained after sampling OD data by SMOTE. It can be seen from Fig 2: the problem of imbalance of accident categories is basically solved after all kinds of accident groups are sampled.

**Fig 3:**
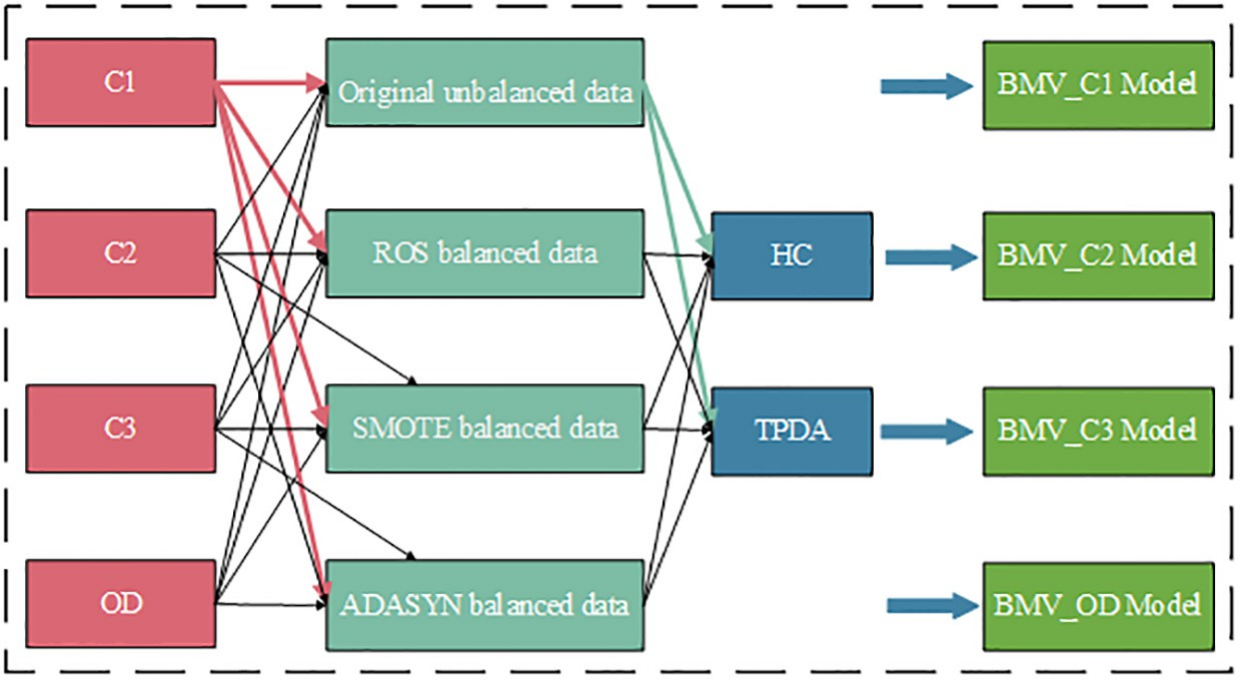
Construction of BN models in different accident clusters.

In this study, different Bayesian classifiers are developed based on the treated and untreated datasets, and five measures are employed for the performance evaluation. To obtain the best BN model for each type of accident cluster, four data (including one unprocessed original unbalanced data, and three sampled and processed balanced data) of each accident cluster were combined with TPDA and HC. All BN models were then generated. Finally, 5-fold cross-validation was utilized to obtain the accuracy, sensitivity, specificity, G-mean, and AUC values of each model, and the optimal BN model for each type of accident cluster was selected based on these results. The optimal BN models for the C1, C2, C3, and OD accident clusters were the BMV_C1, BMV_C2, BMV_C3, and BMV_OD models, respectively. The modeling process is shown in Fig 3.

**Fig 4:**
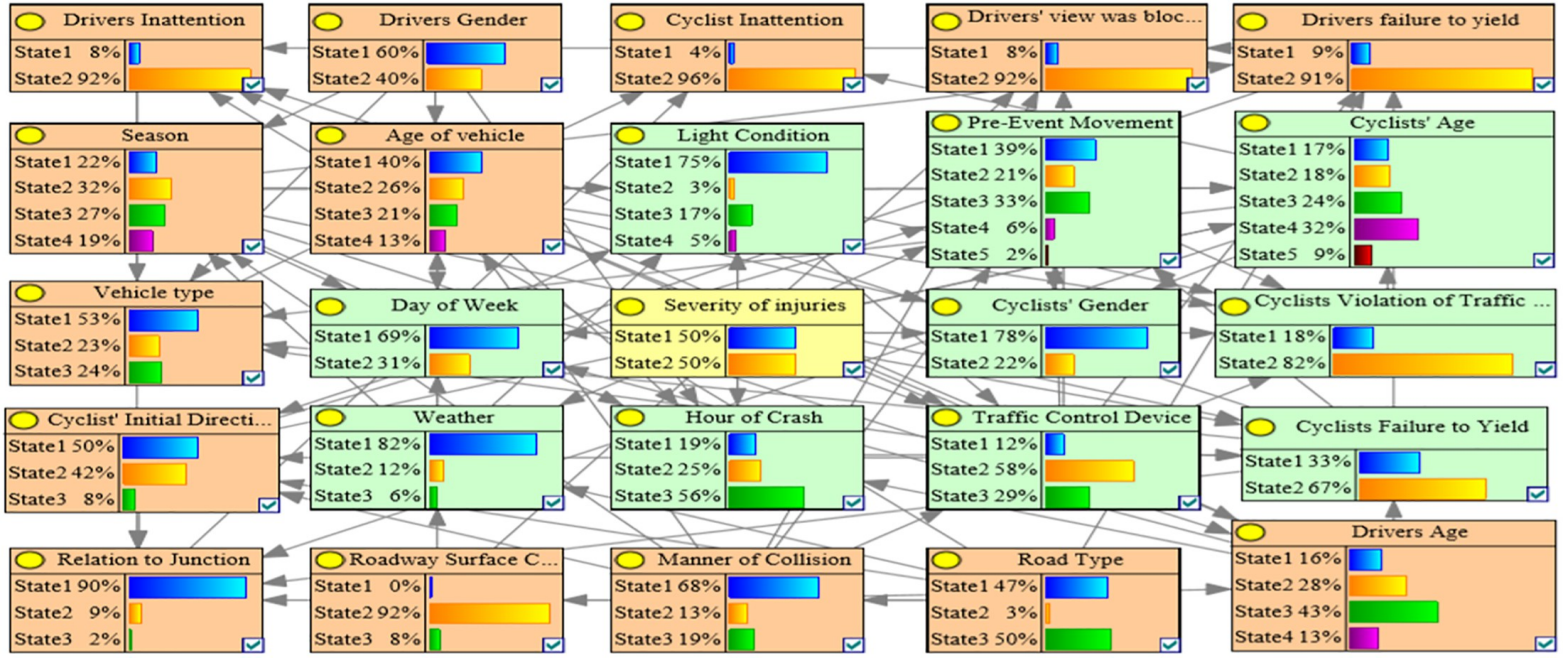
Bayesian network for BMV_C1 model.

**Fig 5:**
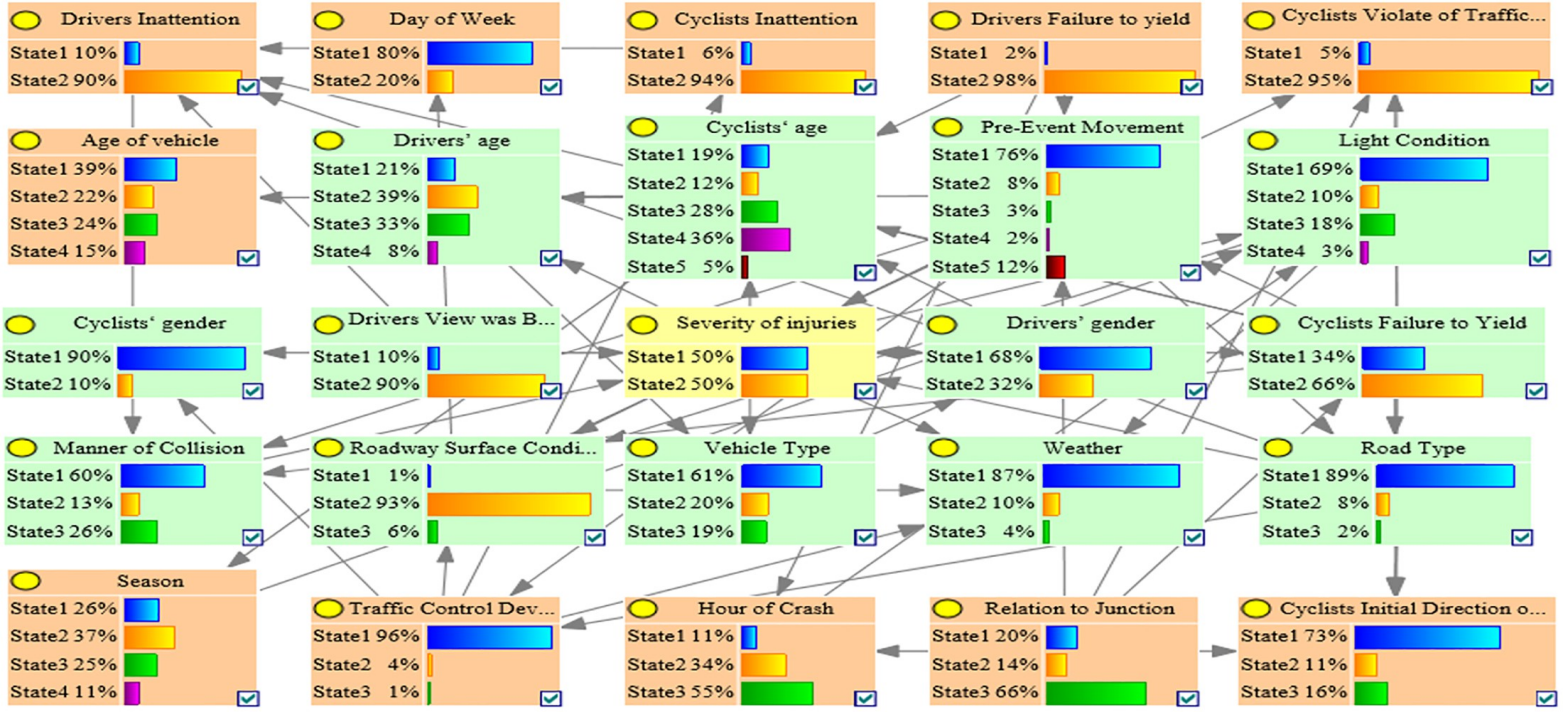
Bayesian network for BMV_C2 model.

**Fig 6:**
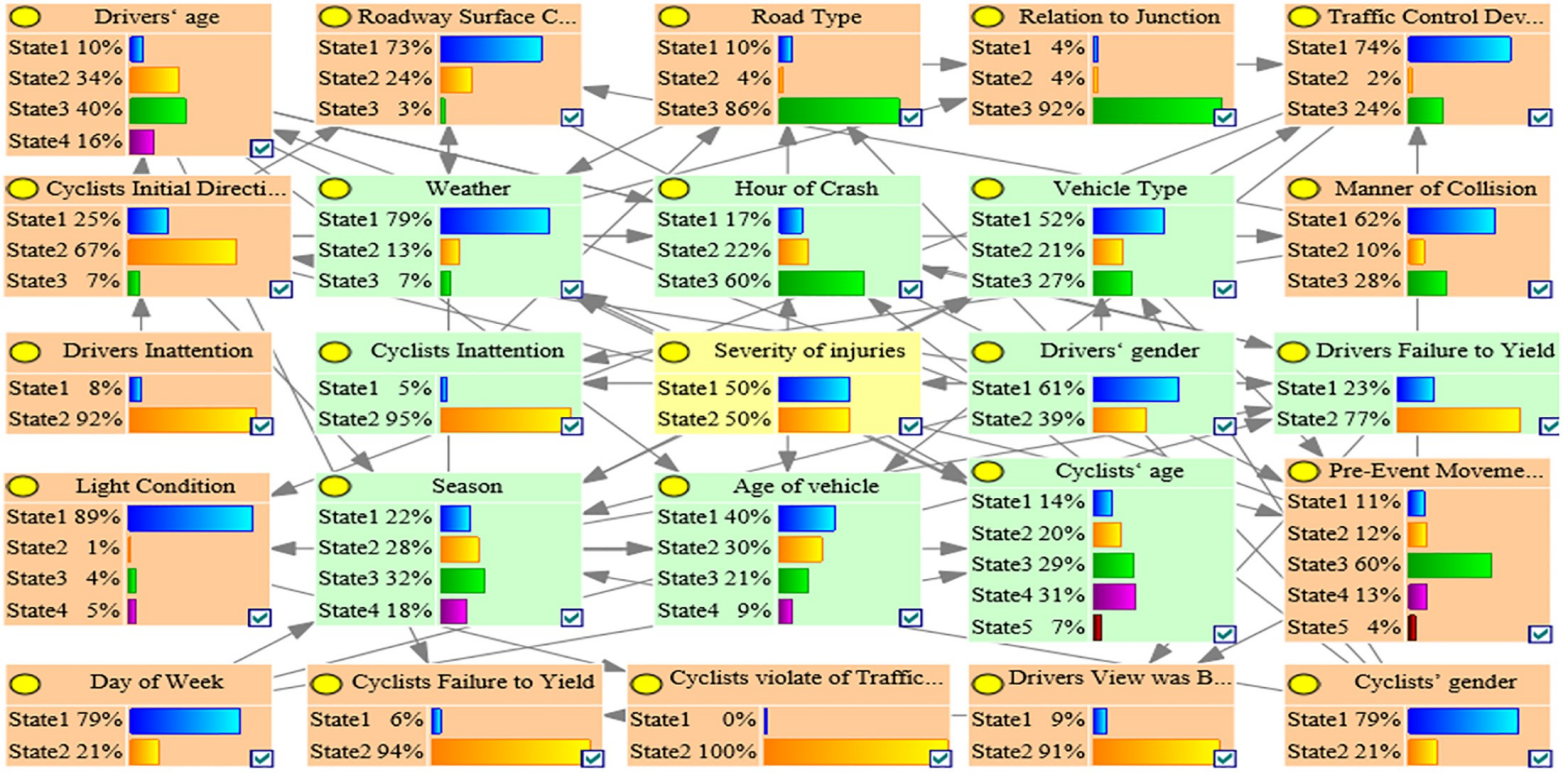
Bayesian network for BMV_C3 model.

**Fig 7:**
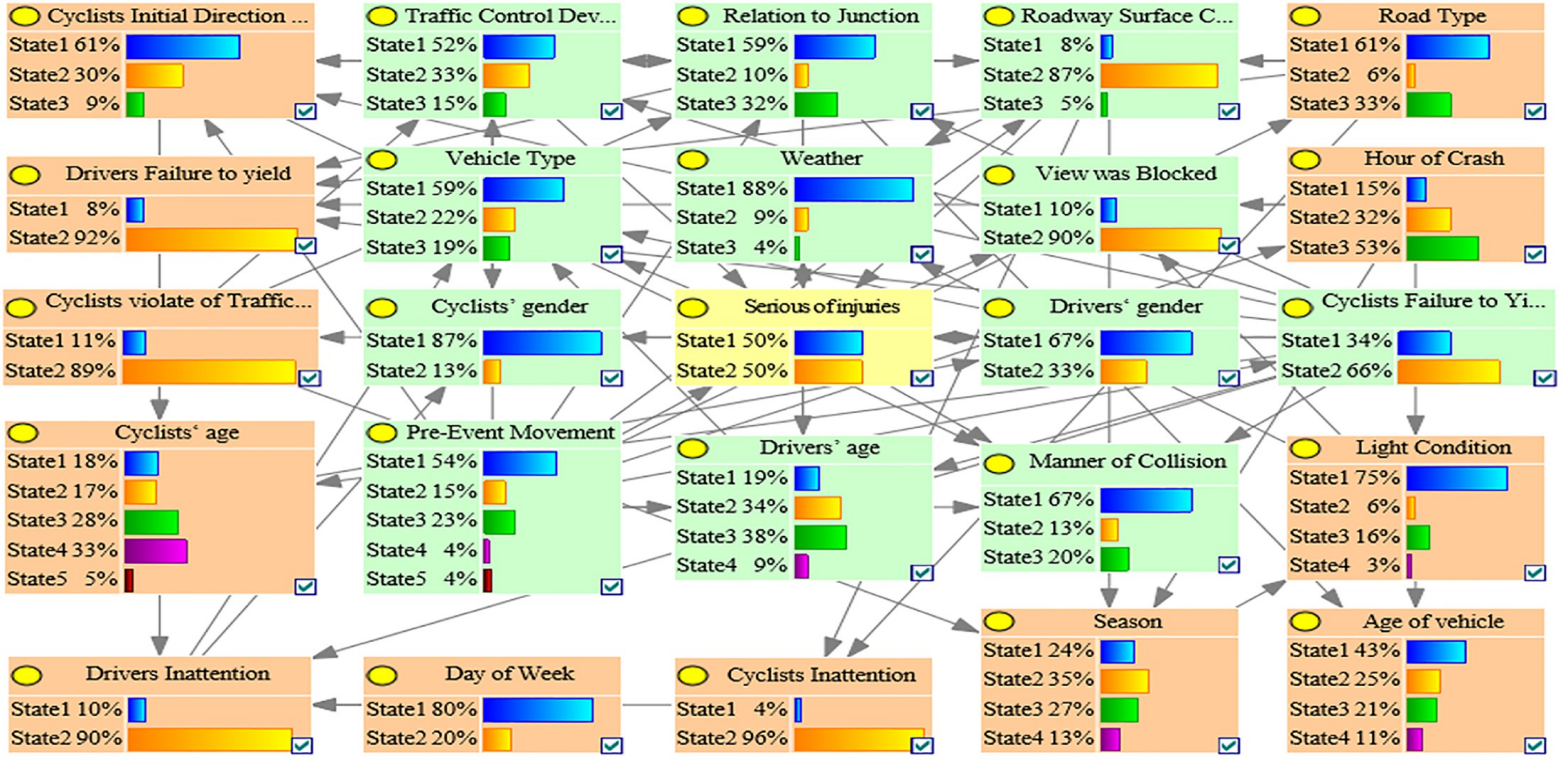
Bayesian network for BMV_OD model.

Because both the G-mean and AUC values represent the comprehensive performance of the model and can be reasonably evaluated even if the data are in an imbalance state, the one with the highest G-mean and AUC value in the C1, C2, C3, and OD accident clusters was used as the optimal BN model. Table 3 shows that the C1_ROS_HC model had the highest G-mean and AUC value in the C1 accident group, which indicated that the C1_ROS_HC model was the optimal BMV_C1 model. Similarly, the C2_SMOTE_TPDA, C3_ROS_HC, and OD_SMOTE_TPDA models were the optimal BMV_C2, BMV_C3, and BMV_OD models, respectively. Tables 3–6 show the various evaluation metrics for different BN models in different accident clusters. Figs 4–7 show the BN plots for the BMV_C1, BMV_C2, BMV_C3, and BMV_OD models.

**Table 3:** Evaluation indexes of different BN models in the C1 accident cluster.

Models	Index				
	*Accuracy*	*Sensitivity*	*Specificity*	*G-mean*	*AUC*
C1_HC	0.907	1.000	0.000	0.000	0.575
C1_TPDA	0.907	1.000	0.000	0.000	0.575
C1_ROS_HC	0.892	0.841	0.943	0.891	0.960
C1_ROS_TPDA	0.848	0.758	0.939	0.843	0.926
C1_SMOTE_HC	0.776	0.763	0.789	0.776	0.858
C1_SMOTE_TPDA	0.828	0.824	0.832	0.828	0.907
C1_ADASYN_HC	0.779	0.763	0.794	0.779	0.854
C1_ADASYN_TPDA	0.829	0.828	0.830	0.829	0.911

**Table 4:** Evaluation index of different BN models in the C2 accident cluster.

Models	Index				
	*Accuracy*	*Sensitivity*	*Specificity*	*G-mean*	*AUC*
C2_HC	0.802	1.000	0.000	0.000	0.577
C2_TPDA	0.801	0.999	0.000	0.000	0.645
C2_ROS_HC	0.708	0.641	0.776	0.705	0.769
C2_ROS_TPDA	0.678	0.599	0.757	0.673	0.755
C2_SMOTE_HC	0.713	0.679	0.746	0.712	0.782
C2_SMOTE_TPDA	0.731	0.707	0.756	0.731	0.811
C2_ADASYN_HC	0.704	0.677	0.730	0.703	0.777
C2_ADASYN_TPDA	0.706	0.680	0.733	0.706	0.774

**Table 5:** Evaluation indexes of different BN models in the C3 accident cluster.

Models	Index				
	*Accuracy*	*Sensitivity*	*Specificity*	*G-mean*	*AUC*
C3_HC	0.937	1.000	0.000	0.000	0.425
C3_TPDA	0.937	1.000	0.000	0.000	0.435
C3_ROS_HC	0.963	0.925	1.000	0.962	0.995
C3_ROS_TPDA	0.916	0.848	0.985	0.914	0.972
C3_SMOTE_HC	0.930	0.910	0.951	0.930	0.973
C3_SMOTE_TPDA	0.943	0.923	0.964	0.943	0.983
C3_ADASYN_HC	0.898	0.889	0.907	0.898	0.955
C3_ADASYN_HC	0.918	0.887	0.950	0.918	0.970

**Table 6:** Evaluation indexes of different BN models in the OD accident cluster.

Models	Index				
	*Accuracy*	*Sensitivity*	*Specificity*	*G-mean*	*AUC*
OD_HC	0.879	1.000	0.000	0.000	0.615
OD_TPDA	0.879	1.000	0.000	0.000	0.617
OD_ROS_HC	0.681	0.627	0.735	0.679	0.741
OD_ROS_TPDA	0.685	0.638	0.732	0.683	0.757
OD_SMOTE_HC	0.678	0.660	0.697	0.678	0.740
OD_SMOTE_TPDA	0.743	0.730	0.755	0.742	0.817
OD_ADASYN_HC	0.701	0.655	0.746	0.699	0.757
OD_ADASYN_TPDA	0.702	0.662	0.741	0.700	0.774

### 5.3 Key elements of the BMV BN model

**Fig 8:**
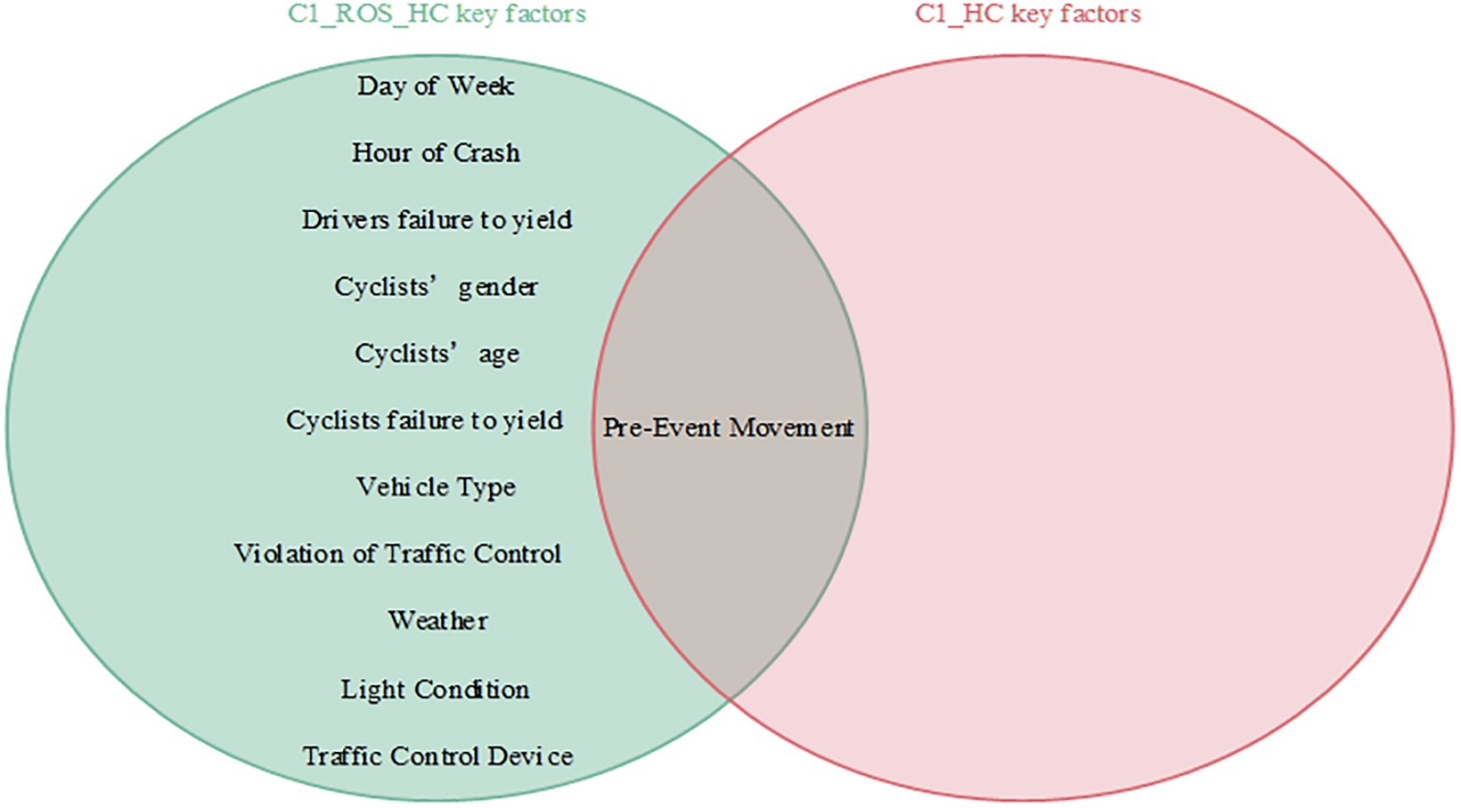
Venn diagram of key factors in the C1_ROS_HC model and key factors in the C1_HC model.

**Fig 9:**
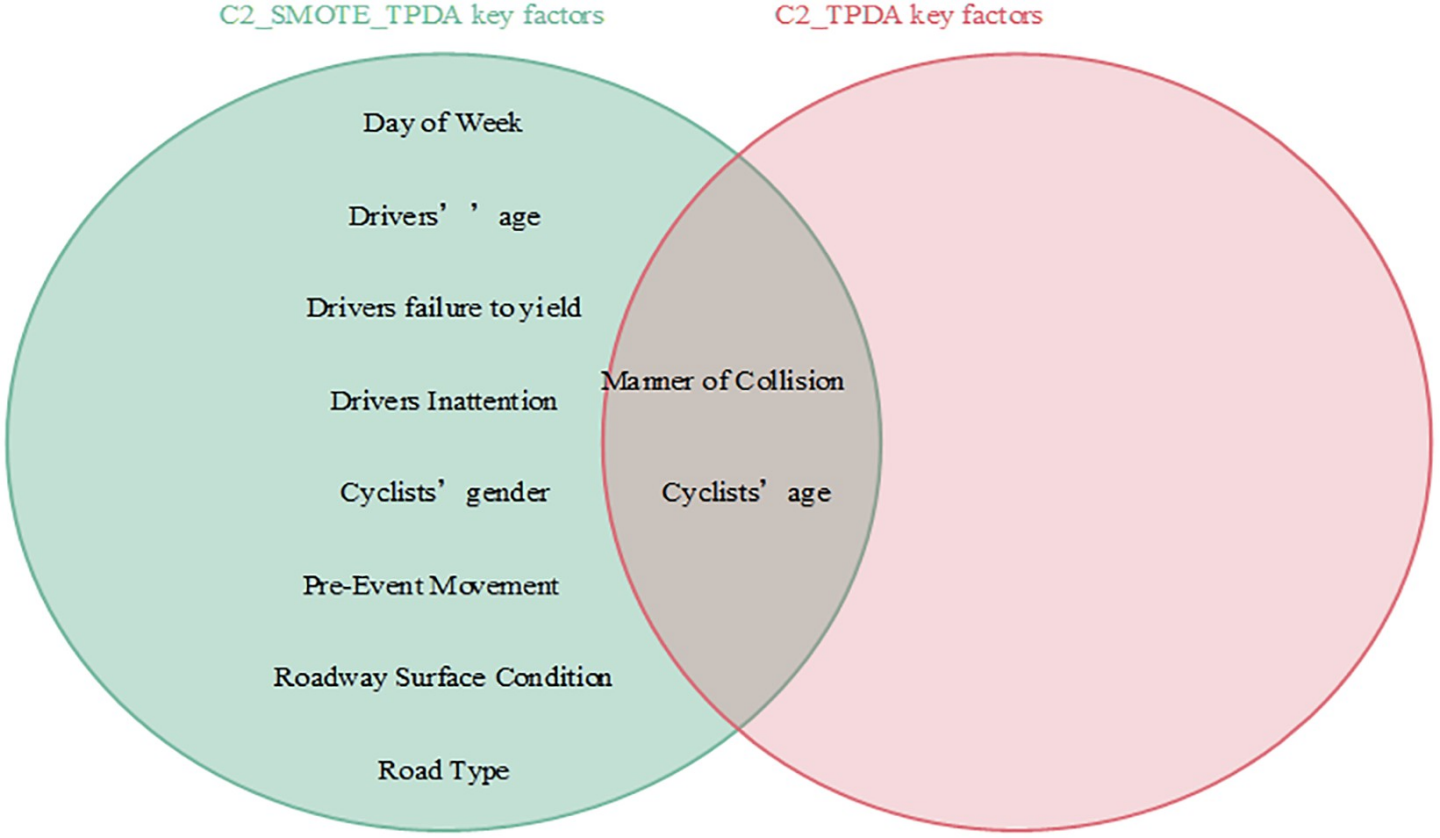
Venn diagram of key factors in the C2_SMOTE_TPDA model and key factors in the C2_TPDA model.

**Fig 10:**
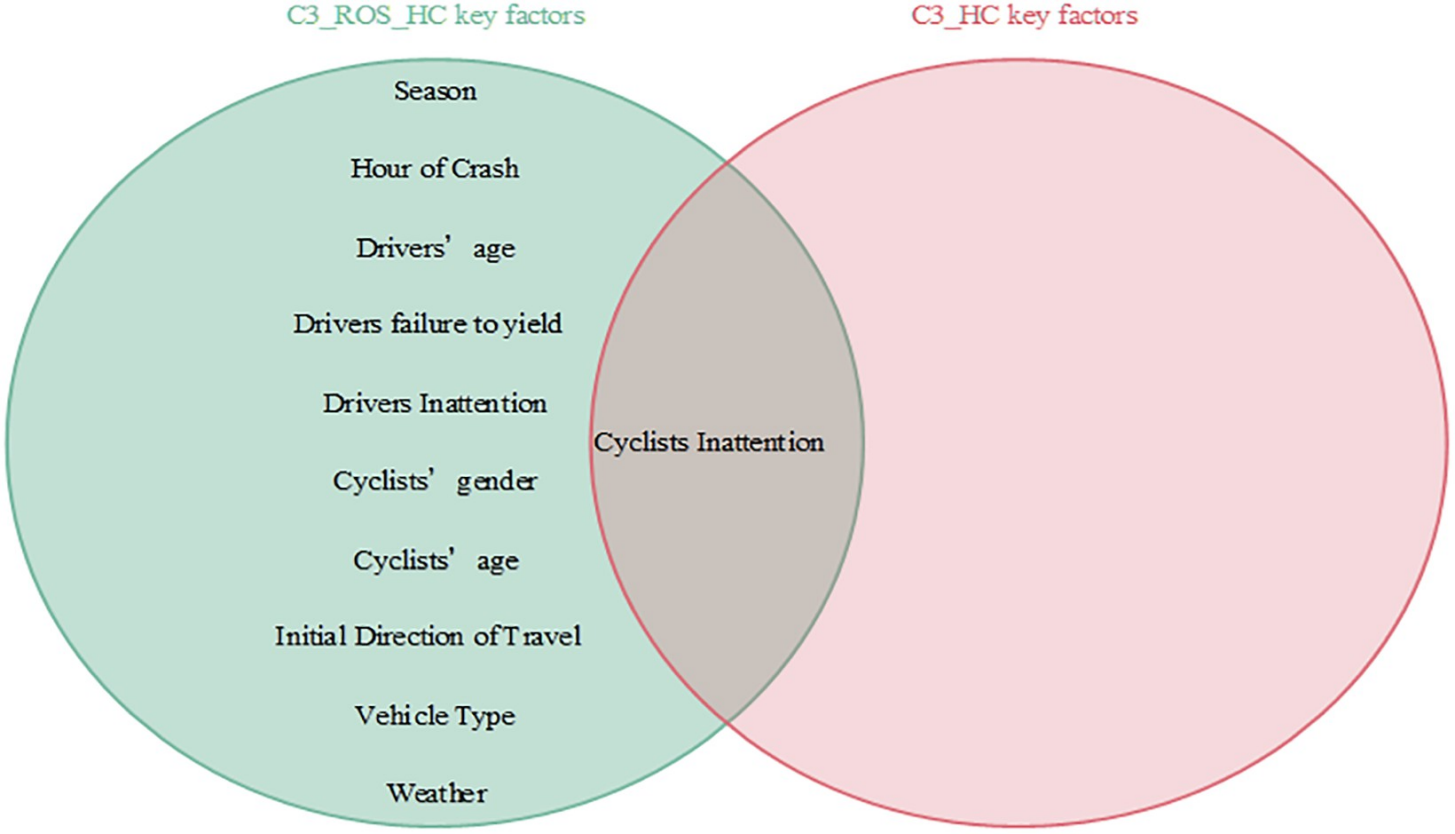
Venn diagram of key factors in the C3_ROS_HC model and key factors in the C3_HC model.

**Fig 11:**
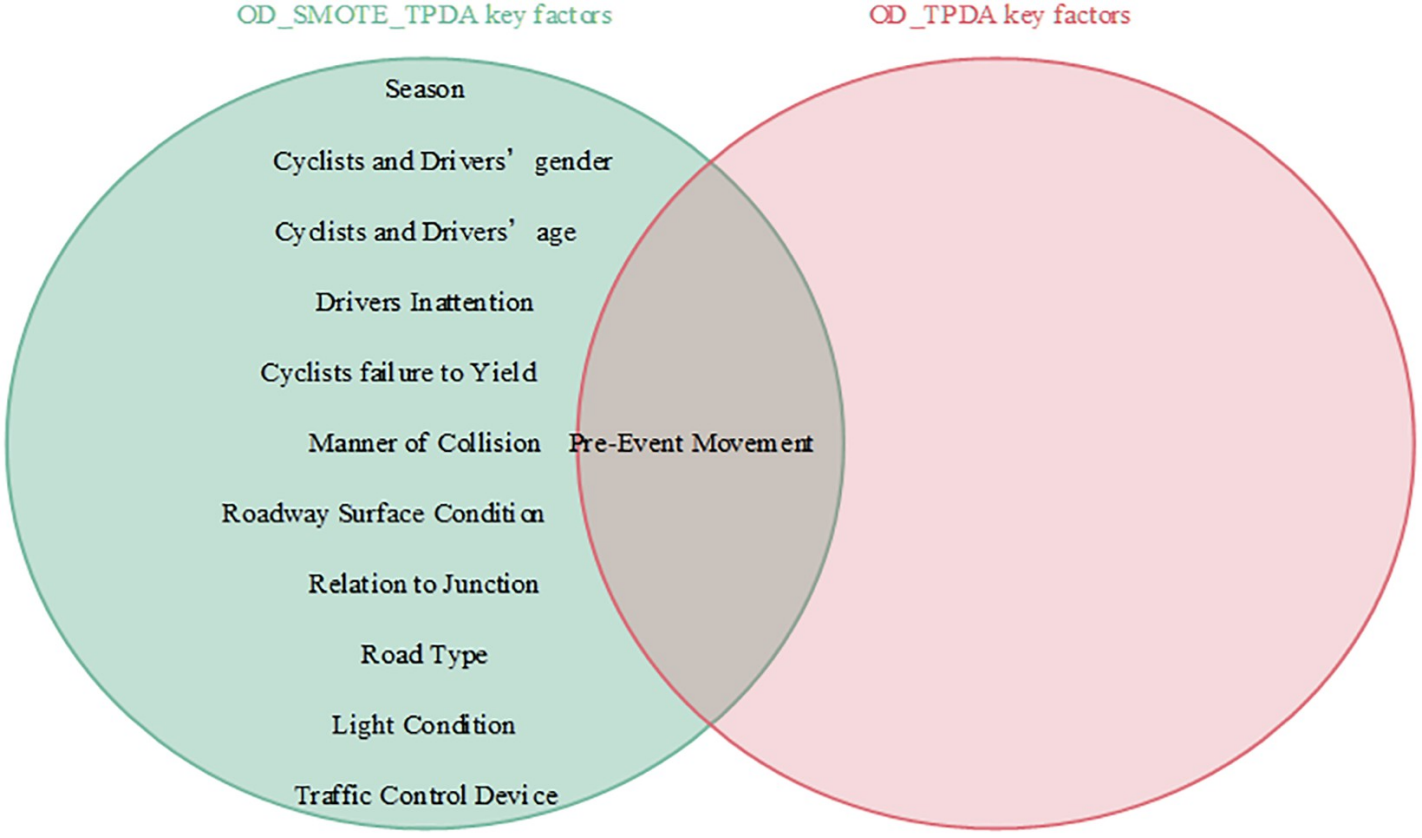
Venn diagram of key factors in the OD_SMOTE_TPDA model and key factors in the OD_TPDA model.

Interdependencies between different variables can be easily established based on the BN theory. Similar to other researchers studying traffic accidents, we are more interested in the key factors that influence the serious injuries (both Serious Injury and Fatal Injury) to cyclists. Thus, the present study focused only on the factors that have a direct dependence on serious injuries and are considered as the key factors that influence serious injuries to cyclists [7]. Figs 8–11 show the key factors affecting the serious injuries of cyclists in the BMV_C1, BMV_C2, BMV_C3, and BMV_OD models, respectively, as well as the key factors affecting the serious injuries to cyclists in the BN model constructed based on the same BN algorithm but without the balanced treatment algorithm.

To investigate which values of the key factors contribute to the probability of a cyclist sustaining a serious injury (hereinafter referred to as the "serious injury rate"), based on the optimal BN model of different accident groups, each value of each key factor is used as "evidence" (i.e., the probability of occurrence of one value of the variable is set to 100% and the probability of occurrence of the rest of the values is set to 0%) in GeNIe 4.0, and the serious injury rate of each node under the evidence is observed. Based on the optimal BN model for different accident clusters, each value of each key factor is used as "evidence" in GeNIe 4.0 software (i.e., the probability of occurrence of a certain value of the variable is set to 100%, and the probability of occurrence of the rest of the values is set to 0%), and the serious injury rate of the seriously injured node is observed under each evidence.

**Fig 12:**
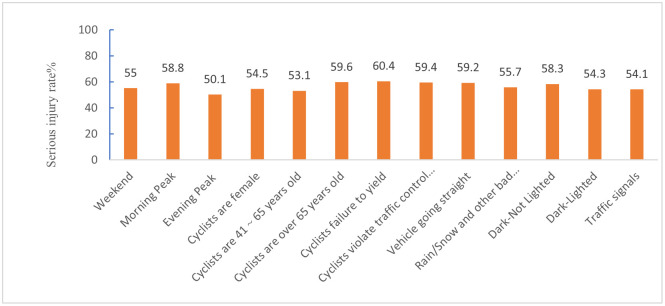
Values for all key factors in the C1_ROS_HC model for which serious injury rates were higher than non-serious injury rates.

**Fig 13:**
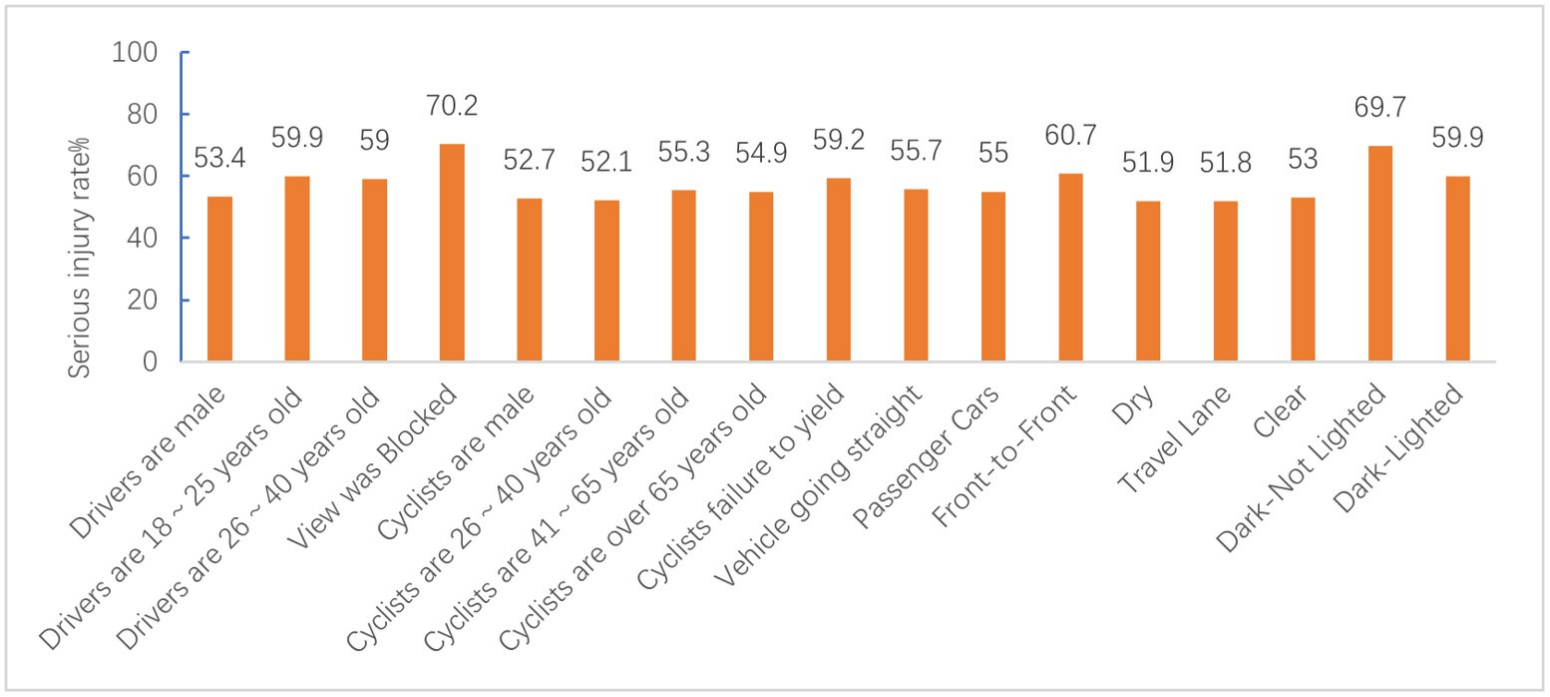
Values for all key factors in the C2_SMOTE_TPDA model for which serious injury rates were higher than non-serious injury rates.

**Fig 14:**
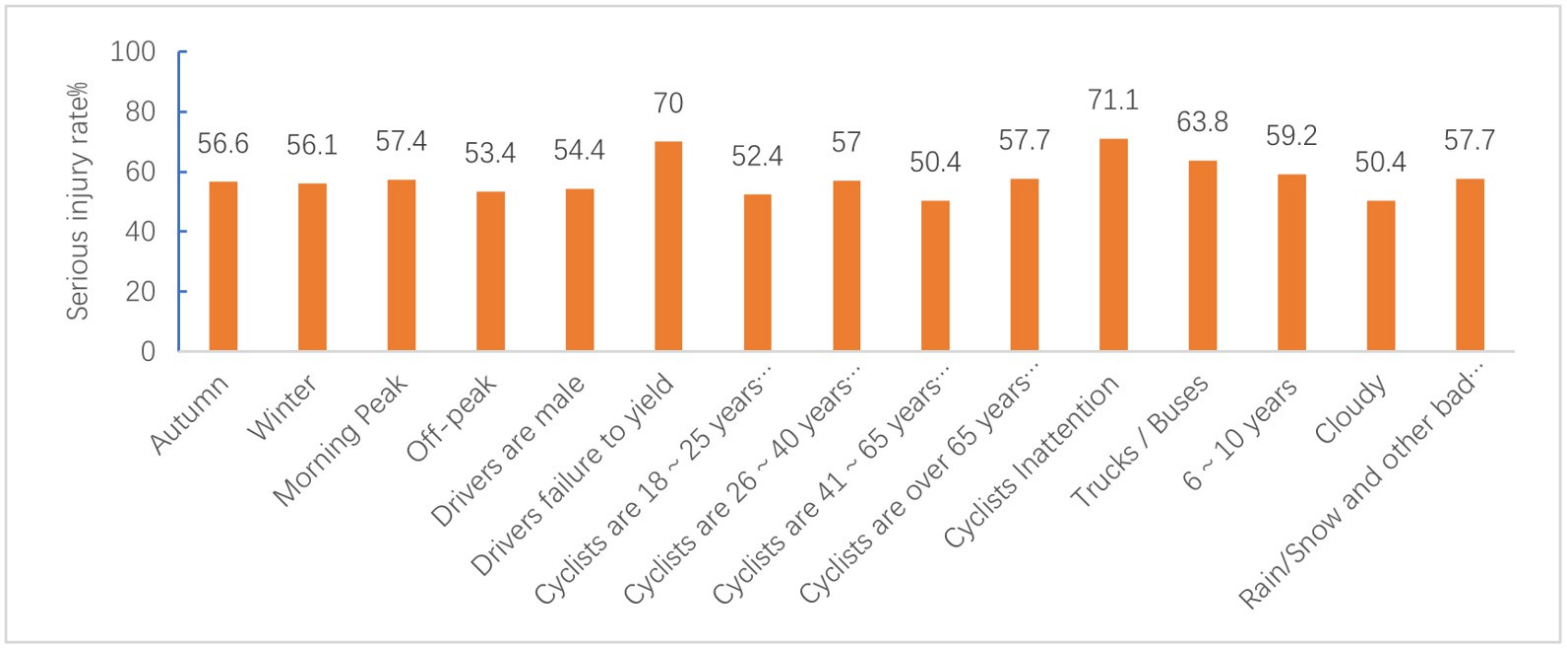
Values for all key factors in the C3_ROS_HC model for which serious injury rates were higher than non-serious injury rates.

**Fig 15:**
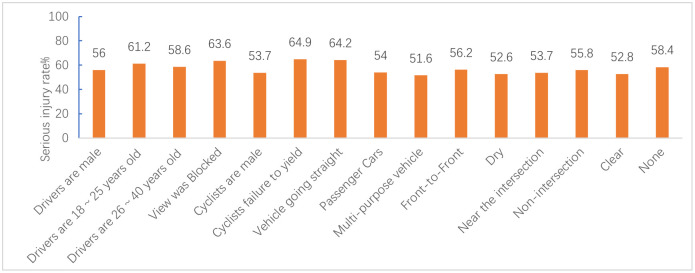
Values for key factors in the OD_SMOTE_TPDA model for which serious injury rates were higher than non-serious injury rates.

Figs 12–15 show the values of all key factors in the BMV_C1, BMV_C2, BMV_C3, and BMV_OD models for which the serious injury rate was higher than the non-serious injury rate.

## 6 Discussion

### 6.1 Analysis of key factors in the BMV model

#### 6.1.1 Time of crash characteristics

In the C1 accident cluster, accidents occurring on weekends and in the morning peak increased the rate of serious injuries to cyclists by 5% and 8.8%, respectively. This is consistent with Kim JK’s [34] study that both drivers and cyclists during rush hour tend to drive and cycle more aggressively, resulting in severe injuries to cyclists.

In the C3 accident cluster, accidents occurring in the fall and winter seasons increased the rate of serious injury to cyclists by 6.6% and 6.1%, respectively, which was similar to previous studies [35]. Similar to the C1 accident cluster, accidents occurring in the morning peak increased the rate of serious injury to cyclists by 7.4%, but unlike the C1 accident cluster, accidents occurring in the off-peak hours also increased the rate of serious injury to cyclists by 3.4% in the C3 accident cluster. Combined with the characteristics of the C3 accident cluster. The potential reason is that most of the accidents in the C3 accident cluster occur on dedicated feeder roads, i.e., roads and intersections where commercial centers, residences, parking lots, etc., enter arterials, which have little to do with peak hours.

#### 6.1.2 Driver and cyclist characteristics

In the C1 accident cluster, female cyclists were more likely to be seriously injured compared to male cyclists, which may be explained by 99% of the C1 accident clusters occurring at and near intersections. Some studies have shown that female cyclists are more likely to be involved in dangerous conflicts at intersections than male cyclists [36]. In particular, cyclists’ failure to yield and traffic control device violations increased the rate of serious injuries to cyclists by 10.4% and 9.4%, respectively. Moreover, older cyclists were more likely to be seriously injured, with a 9.6% increase in the probability of serious injury for cyclists over 65 years of age. The potential reason may be due to decreases in physical fitness and hazard perception that occur with age, and injuries tend to be more severe in collisions.

In the C2 accident cluster, failure to yield to cyclists increased the rate of serious injuries by 9.2%. Moreover, drivers aged 18 to 25, drivers aged 26 to 40, and male drivers increased the rate of serious injuries to cyclists by 9.9%, 9%, and 3.4%, respectively. These results may be attributed to the majority of accidents in this accident cluster occurring on non-intersection general roadways, and drivers aged 18 to 40 years and male drivers drive more aggressively and faster on general roadways, leading to more serious accidents [37]. Unlike the C1 accident cluster, the C2 accident cluster had a 2.7% increase in the probability of serious injuries for male cyclists. The reason for this may be that most of the C2 accident clusters occurred on common non-intersection roadways with better riding conditions, and male cyclists are more likely to engage in risky riding behaviors than female cyclists when traveling on this roadway [35]. Of note, there is no reliable conclusion on the effect of cyclist gender on injury severity. Li et al. [38] concluded that male cyclists have more driving experience and are calmer than female in t accidents, which indicates that female cyclists are more likely to cause serious injuries in accidents. Therefore, further research is needed to clarify these inconsistent findings. Finally, an obstructed field of view of the driver increased the serious injury rate of cyclists by 20.2%, which agreed with the findings of previous studies on BMV accidents.

In the C3 accident clusters, male drivers increased the probability of serious injury to cyclists by 4.4%, and driver failure and distracted cyclists increased the rate of serious injury to cyclists by 20% and 21.1%, respectively. The majority of accidents in the C3 accident cluster involved cyclists about to exit a dedicated slip road, and 81.19% of C3 accident clusters did not have traffic control devices. Because slip roads are narrower than normal intersections, drivers traveling on the main road before the accident are less likely to notice cyclists.

#### 6.1.3 Vehicle characteristics

In the C1 and C2 accident clusters, the vehicle travel status straight ahead increased the serious injury rate of cyclists by 9.2% and 5.7%, respectively, which may have been attributed to the higher impact energy from the collision due to the faster speed of the straight-ahead vehicle compared to the steering and start/stop states.

In the C2 accident clusters, passenger cars increased the serious injury rate of cyclists by 5% because most of the crashes in this cluster occurred at non-intersections where there are no blind spots in the field of view. The speed of passenger cars at non-intersections is fast, causing both the cyclist and the driver to have time to avoid the crash before the crash. The rate of serious injury to cyclists increased by 10.7% when the collision location was at the front of the car. One possible explanation is that bicycles are more like to crash in frontal collisions, resulting in more serious accidents, especially on non-intersection roadways where traffic speeds are higher [35]. This type of accident may be addressed by improving the active and passive safety technology of vehicles, such as automatic emergency braking (AEB) systems and hood pop-up technology, and cyclists are also required to wear helmets and protective devices [39].

In the C3 accident cluster, the rate of serious injuries to cyclists increased by 13.8% when the vehicle type was a truck/bus, which was due to the large mass of the truck/bus and the kinetic energy of the vehicle before the crash. Driving age of 6 to 10 years caused an increase in the probability of serious injury to cyclists. These results may be explained by drivers with 6 to 10 years of driving experience having a higher threshold of risk acceptance and are instead prone to overconfidence and overdriving behavior.

#### 6.1.4 Road characteristics

The results of the C2 accident cluster indicated that the probability of a cyclist being seriously injured while riding on dry pavement increased by 1.9%, and the probability of a cyclist being seriously injured while riding in the carriageway increased by 1.8%. In contrast, the probability of being seriously injured while riding in the bike lane/shoulder decreased. Thus, these findings suggest that roadways should be designed to achieve as much separation as possible between the machine and non-machine as well as to arrange machine and non-machine separation zones.

#### 6.1.5 Environment attributes

In the C1 accident cluster, the rate of serious injuries to cyclists increased by 5.7% when accidents occurred in bad weather, such as rain/snow, which is attributed to rain/snow reducing visibility, ultimately affecting the risk perception and reaction ability of drivers and cyclists. In particular, some studies have also shown that bad weather, such as rain/snow, reduces the probability of cyclist injury or death [36], which is attributed to a risk compensation effect mechanism [38]. The absence of lighting at night increased the probability of serious injury by 8.3%, and the probability of serious injury increased by 4.3% even with lighting at night, which is due to the lack of light at night and the fact that most bicycles do not have lights similar to electric two-wheelers and are not easily detected by drivers. In contrast to most studies, the present study found that when the traffic control device was a traffic signal, it increased the rate of serious injuries to cyclists by 4.1%. Moreover, 91.13% of the accidents in the C1 accident cluster occurred at intersections. It has been noted that the probability of serious injury increases when traffic signal lights are present at intersections, which may be due to high traffic flow, leading to an increased risk for cyclists [40].

In the C2 accident cluster, clear weather increased the probability of serious injury to cyclists by 3% due to similar reasons as those for C2 accidents occurring at non-intersections where drivers are more likely to speed in clear weather. Similar to the findings for the C1 accident cluster, both no lighting at night and lighting at night increased the probability of serious injury to cyclists in the C2 accident cluster.

In the C3 accident cluster, inclement weather, such as rain/snow, increased the probability of serious injury to cyclists. Combining the three accident clusters showed that bad weather, such as rain/snow, increased the probability of serious injury accidents at intersections and dedicated branch intersections, while sunny days increased the probability of serious injury accidents at non-intersection general roads. These findings confirmed the heterogeneity of weather condition factors, further explaining why weather effects are controversial in previous studies on BMV accidents.

There were additional similarities between the OD accident cluster and the three sub-accident clusters, but these similarities were not further analyzed. Unlike the three sub-accident clusters, roadway type was only found to be a key factor in the OD accident cluster, and the results showed that accidents occurring near intersections or at non-intersections increased the probability of serious injury to cyclists by 3.7% and 5.8%, respectively. These findings were consistent with the majority of previous studies showing that non-intersection roadways are more dangerous than intersection roadways. These results suggested that the division of the entire data into three accident clusters by LCA explains the heterogeneity of roadway types in the OD accident clusters.

### 6.2 Heterogeneity analysis

We next evaluated the differences in critical factors for different accident clusters (Table 7).

**Table 7:** Key factors for different accident clusters.

Key factors		Accident clusters			
		OD	C1	C2	C3
Crash characteristics	Season				*
	Day of Week		*		
	Hour of Crash		*		*
Drivers characteristics	Gender	*		*	*
	Age	*		*	
	Failure to yield				*
	View was Blocked	*		*	
Cyclists characteristics	Gender	*	*	*	
	Age		*	*	*
	Failure to yield	*	*	*	
	Violation of Traffic Control Device		*		
	Inattention				*
Vehicle characteristics	Pre-Event Movement	*	*	*	
	Vehicle Type	*		*	*
	Manner of Collision	*		*	
	Age of vehicle				*
Roadway characteristics	Roadway Surface Condition	*		*	
	Relation to Junction	*			
	Cross Section			*	
Environmental characteristics	Weather	*	*	*	*
	Light Condition		*	*	
	Traffic Control Device	*	*		

Note: where "*" means that the factor is a critical factor for a specific accident cluster.

A comparative analysis of the key factors for the OD incident cluster and the three sub-accident clusters in Table 7 revealed four meaningful results.

First, some factors were identified as critical only in sub-accident clusters but not in the OD accident cluster. For example, accident week and cyclists’ violation of traffic control device were identified as critical factors only in the C1 accident cluster, in which the accident occurring on a weekend and the cyclist failing to yield increased the probability of serious injury to the cyclist by 5% and 9.4%, respectively. In addition, roadway cross-section type was a key factor only in the C2 accident cluster, in which cyclists riding in the carriageway (motorway) increased the probability of serious injury to cyclists and riding in the bike lane/shoulder (non-motorway) reduced the probability of serious injury to cyclists. Similarly, season, driver failure to yield, distracted cyclist riding, and age of driving were key factors only in the C3 accident cluster. These findings reaffirmed the necessity and importance of using LCA to classify accident data into homogeneous sub-data, which not only explores the key factors hidden in OD accident clusters to provide more comprehensive information but also provides insight into the differences of key factors in different accident clusters to reveal the heterogeneity of the data. Moreover, most of the BN models constructed based on homogeneous sub-data outperformed the BN models constructed based on OD accident clusters in terms of G-mean and AUC values (Section 5.3).

Second, some factors were identified as key factors in both OD accident clusters and sub-accident clusters. For example, the driver’s gender and vehicle type were key factors in the OD, C2, and C3 accident clusters, while the cyclists’ gender, cyclists’ failure to yield, and pre-event movement were key factors for the OD, C1, and C2 accident clusters. In addition, some factors were identified as key factors only in the OD accident cluster and a specific class of sub-accident clusters. For example, the driver’s age, the view was blocked, and the crash location were only critical factors for OD accident clusters and C2 accident clusters. For these factors, the use of LCA helps to clarify their influence patterns on bicycles and accidents, providing the basis for sub-models to explain in more detail the factors influencing serious injuries to cyclists.

Third, some of the factors were identified as key factors in all three sub-accident clusters, but their effects on cyclist injury severity were not consistent and even sometimes opposite. In the C1 accident cluster, "female" cyclists were more likely to be seriously injured than male cyclists, whereas male cyclists were more likely to be seriously injured than female cyclists in the C2 accident cluster. In the C1 and C3 accident clusters, rain/snow and other bad weather cyclists were more likely to be seriously injured, whereas sunny weather cyclists were more likely to be seriously injured than female cyclists in the C2 accident cluster. In the C1 accident cluster, cyclists were less likely to be seriously injured during "off-peak" hours, while cyclists were more likely to be seriously injured during off-peak hours in the C3 accident cluster.

Fourth, although the use of LCA to divide the overall accident data into homogeneous sub-data revealed some new factors, several key factors of the OD accident clusters may have been overlooked, i.e., some of the factors were key factors in the OD accident clusters but not in the sub-accident clusters. For example, roadway type was a key factor only in the OD accident cluster, in which cyclists were vulnerable to serious injury at non-intersections and near intersections but not at intersections vulnerable to non-serious injuries.

### 6.3 Analysis of data before and after sampling

Compared to the BN model constructed based on unbalanced data, the BN model constructed based on balanced data did not show significant improvement in overall correctness and sensitivity but had substantial improvement in Specificity, G-mean, and AUC values (Tables 3–6). This phenomenon may be explained because machine learning methods inherently assume that the data are balanced and subject to the dominant effect of the majority class, and models constructed based on the original unbalanced data will be biased toward the majority class in terms of prediction results, resulting in poor classification accuracy or even invalid models for the minority class. For example, the overall correctness and sensitivity of the C1_HC model and C1_TPDA model were higher than 0.9, but the specificity index was 0, i.e., the classification accuracy of the minority class was 0. This type of classifier is called a foolish classifier. Thus, these findings illustrated that the overall correctness index cannot represent the validity of the model, indicating that the sensitivity and specificity indexes need to be considered.

With the same BN algorithm, the BN model constructed based on the balanced dataset identified more key factors affecting serious injuries (Figs 8–11). For example, the week of the accident, time period, cyclists’ gender, cyclists’ age, cyclists’ failure to yield, cyclists’ violation of traffic control devices, vehicle driving status, weather conditions, lighting conditions, and traffic control devices (a total of 10 key factors) were identified as critical factors in the C1_ROS_HC model constructed based on the balanced dataset. Some researchers have found that these factors influence serious injuries to cyclists [20]. However, only vehicle driving status was found to be a critical factor in the C1_HC model constructed based on the unbalanced data set. These findings suggested that the categories of accident data in an unbalanced state may mask the normal relationship between influencing factors and injury severity, leading to misleading research findings. Therefore, these results indicated that appropriate data balancing techniques must be used to reduce the effects of unbalanced category data.

### 6.4 Suggestions on prevention and control of bicycle accidents

**Fig 16:**
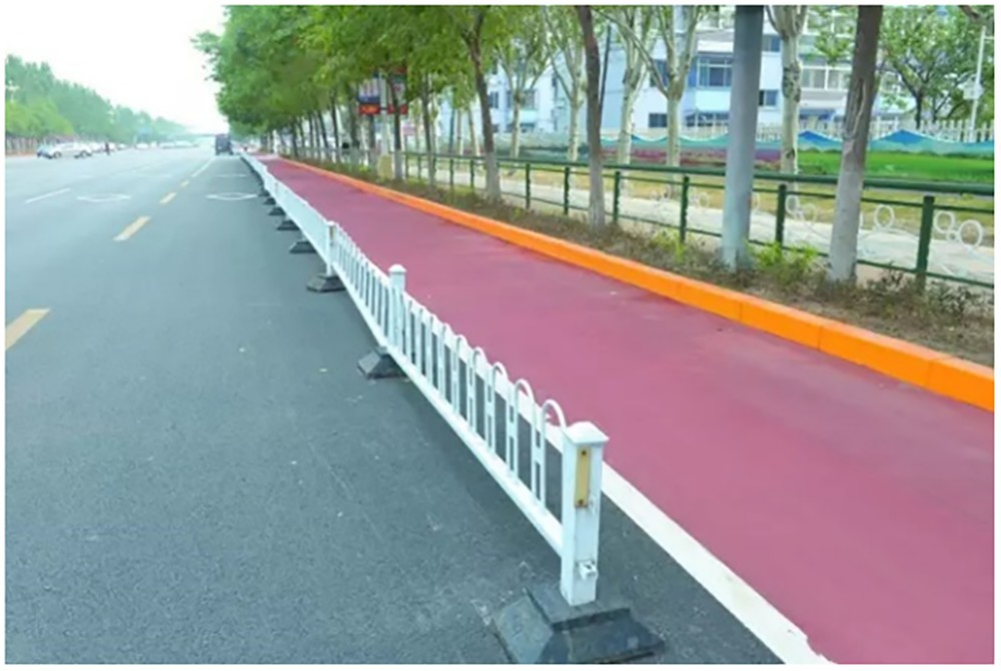
Colored non-motorized lanes.

**Fig 17:**
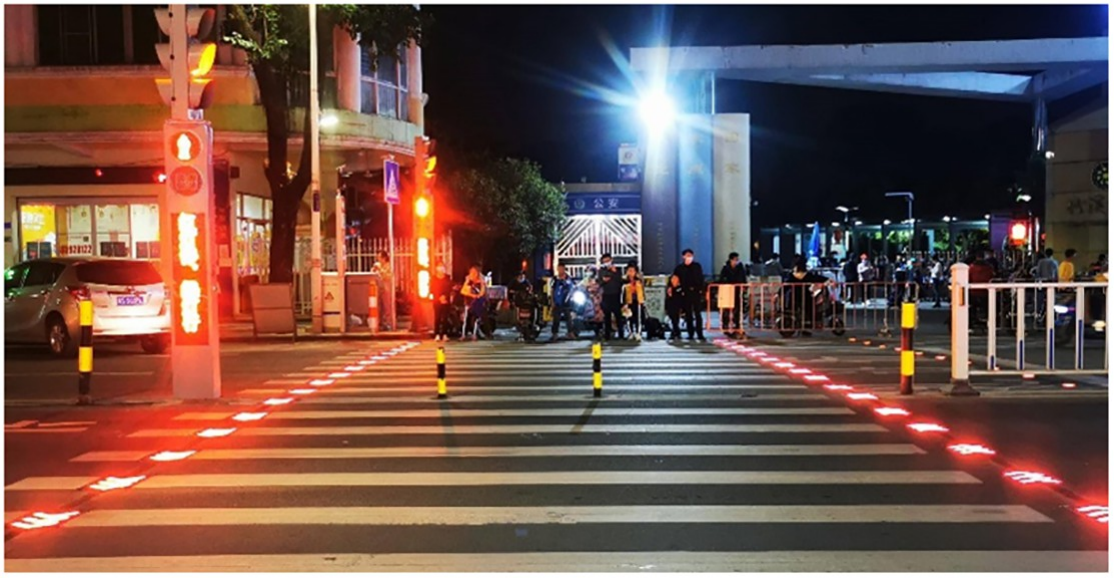
Intelligent zebra crossing.

Based on the above analysis of different accident groups, this section puts forward targeted prevention and control recommendations for bicycle accidents and different accident groups from four aspects: people, vehicles, roads, and environment:

1. Prevention and control recommendations for people
  ①Improve the safety awareness of drivers and cyclists and reduce the occurrence of unsafe behaviors. During off-peak hours at intersections or in bad weather conditions such as rain/snow, it is especially important to prevent the behaviors of "cyclists failing to yield the right-of-way" and "cyclists violating traffic control devices"; and prevent the behaviors of "male" cyclists failing to yield the right-of-way at non-intersections. male" cyclists failing to yield and "male drivers aged 26–40" speeding on non-intersections; and "male drivers failing to yield" and "distracted cyclists" on dedicated slip roads. ②Optimize the driving test system and make the test more difficult. Driving simulators can be used to increase the form of "emergency preparedness test" and "night driving test". ③With the emergence of the aging problem, it is necessary to publicize and encourage the elderly over 65 years old to use free public transportation.
2. Suggestions for prevention and control of vehicles
  ① Enhance the safety inspection of vehicles, especially the vehicle braking system, lighting system and other components, to ensure that the vehicles meet the requirements in terms of safety. ② Enhance the active and passive safety technologies of automobiles to improve the safety performance of automobiles. ③ Allow trucks/buses to make appropriate vehicle modifications, such as adding additional mirrors or reflectors to reduce the impact of blind spots in the field of vision.
3. Prevention and control suggestions for roads
  ① Improve the planning and design of roads. From the analysis of the C2 accident group, it can be seen that cyclists driving in the motor vehicle lane will increase the probability of cyclists being seriously injured, therefore, in the road design and planning, non-intersections should try to avoid mixing of motor vehicle and non-motorized traffic and try to set up "colored non-motorized lanes" as shown in Fig 16. to maximize the attention of motorists and reduce traffic conflicts. ②Enhance the roadway maintenance and safety hazard investigation. Focus on roadway drainage at intersections and dedicated side streets, and promptly clean roadways with standing water or snow. ③ Appropriately increase the roadway’s fault tolerance. Cyclists and drivers will inevitably make mistakes, in the road design can be appropriate to set aside some space for correcting errors, such as appropriate widening of the road surface, from the objective level to increase the reaction time of drivers and cyclists. ④ In the morning rush hour and weekend hours, the focus should be on the driving safety of intersection sections, while in the fall and winter seasons and off-peak hours, the focus should be on the driving safety of dedicated feeder roads.
4. Suggestions for prevention and control of the environment
  ①Improve lighting conditions on roads and strengthen the management of lighting facilities. From the analysis of influencing factors, it can be seen that serious injuries are likely to occur in the environment without lighting at night. To address this problem, we can increase the number of street lamps and improve the coverage and brightness of street lamps in accident-prone areas. ② Optimize transportation infrastructure. As nighttime travel or inclement weather such as rain/snow can affect drivers’ ability to see the crosswalk markings, "smart crosswalks" as shown in Fig 17. It can be popularized near intersections and dedicated branch intersections.③ Enhance the warning and reminder of bad weather.

## 7 Conclusions

In the current paper, we propose a two-stage method integrating LCA, resampling, and BN to investigate the effects of different influencing factors on the injury severity of bicyclists in bicycle accidents when the data are heterogeneous and unbalanced, and the key factors are mined from the characteristics of the time of the accident, the characteristics of the driver and the bicyclist, the characteristics of the vehicle, and the characteristics of the roadway to analyze the heterogeneity, and the results show that:

1. In the C1 accident group "BMV accidents at intersections with traffic control devices", accidents occurring on weekends and in the morning rush hour will increase the serious injury rate of cyclists by 5% and 8.8%, respectively; female cyclists are more prone to serious injuries than male cyclists; the vehicle traveling in a straight line will increase the serious injury rate of cyclists by 9.2%; and the vehicle driving status will increase the serious injury rate of cyclists by 9.2%, respectively. serious injury rate will increase by 9.2%; when the accident occurs in bad weather such as rain/snow, the serious injury rate of cyclists will increase by 5.7%.
2. In accident group C2, "Non-intersection BMV accidents without traffic signal control," failure to yield to a bicyclist increases the rate of serious injury by 9.2%; male bicyclists increase the probability of serious injury by 2.7%; when the vehicle is traveling in a straight line, it increases the rate of serious injury by 5.7%; passenger vehicles increase the rate of serious injury by 5.7%; and when the vehicle is traveling in a straight line, it increases the rate of serious injury by 5.7%.; passenger vehicles increase the rate of serious injury to cyclists by 5%; cyclists are 1.9% more likely to be seriously injured when riding on dry pavement and 1.8% more likely to be seriously injured when riding in a driveway; and sunny days increase the probability of serious injury to cyclists by 3%.
3. In accident group C3, "off-road/private sidewalk BMV accidents without traffic control devices," accidents occurring in the fall and winter seasons increase the rate of serious injury to bicyclists by 6.6% and 6.1%, respectively; a male driver increases the probability of serious injury to a bicyclist by 4.4%, and a driver who fails to comply with the rules, or who is distracted from riding, increases the probability of serious injury to a bicyclist by 1.8%; sunny weather increases the probability of serious injury to a bicyclist by 3%. distracted riding increases the rate of serious injuries to cyclists by 20% and 21.1%, respectively; when the vehicle type is truck/bus, the rate of serious injuries to cyclists increases by 13.8%; and inclement weather such as rain/snow increases the probability of serious injuries to cyclists.

In addition, the results of the key factor heterogeneity analysis showed that female cyclists, inclement weather such as rain/snow, and off-peak were key factors in several subcategories of accident groups, but had opposite effects on the severity of cyclist injuries in different subcategories.

## Supporting information

S1 Dataset (PDF)

S1 Table The complete distribution of featured variables describing cluster characteristics. (PDF)

S2 Table Evaluation index of different BN models in the C1-C3 accident cluster. (PDF)
