# Supplementary material for: Analysis of factors influencing the degree of accidental injury of bicycle riders considering data heterogeneity and imbalance
Source: PLoS One. 2024 May 14;19(5):e0301293. doi: 10.1371/journal.pone.0301293 (PMC11093317; doi:10.1371/journal.pone.0301293)

		LL	BIC(LL)	AIC(LL)	AIC3(LL)	CAIC(LL)	Npar	L²
Model1	1-Cluster	-73255.1	146898.8	146604.2	146651.2	146945.8	47	146510.2
Model2	2-Cluster	-71619.4	144040.7	143432.7	143529.7	144137.7	97	143238.7
Model3	3-Cluster	-70617.7	142450.7	141529.4	141676.4	142597.7	147	141235.4
Model4	4-Cluster	-69946	141520.7	140286	140483	141717.7	197	139892
Model5	5-Cluster	-69428.3	140898.7	139350.7	139597.7	141145.7	247	138856.7
Model6	6-Cluster	-68981.8	140419	138557.6	138854.6	140716	297	137963.6
Model7	7-Cluster	-68643.9	140156.5	137981.7	138328.7	140503.5	347	137287.7
Model8	8-Cluster	-68493.6	140269.3	137781.1	138178.1	140666.3	397	136987.1
Model9	9-Cluster	-68338.9	140373.3	137571.8	138018.8	140820.3	447	136677.8
Model10	10-Cluster	-68249.7	140608.2	137493.3	137990.3	141105.2	497	136499.3
Model11	0-Cluster							10^4

number of clusters

	BIC	AIC	CAIC	Entropy		
1	14.68988	14.66042	14.69458	1	0	146898.8
2	14.40407	14.34327	14.41377	0.962	0.038	144040.7
3	14.24507	14.15294	14.25977	0.9463	0.0537	142450.7
4	14.15207	14.0286	14.17177	0.9197	0.0803	141520.7
5	14.08987	13.93507	14.11457	0.9216	0.0784	140898.7
6	14.0419	13.85576	14.0716	0.891	0.109	140419
7	14.01565	13.79817	14.05035	0.8822	0.1178	140156.5
8	14.02693	13.77811	14.06663	0.8727	0.1273	140269.3
9	14.03733	13.75718	14.08203	0.8621	0.1379	140373.3
10	14.06082	13.74933	14.11052	0.8459	0.1541	140608.2

AIC3
14.66512
14.35297
14.16764
14.0483
13.95977
13.88546
13.83287
13.81781
13.80188
13.79903

df	p-value	Class.Err.	reduction			
3848	6.6e-2794	0				
3798	1.0e-2728	0.038	0.019456	0.021633	0.021285	0.01911
3748	1.2e-2690	0.0537	0.011038	0.01327	0.012913	0.010684
3698	4.4e-2666	0.0803	0.006528	0.008785	0.008423	0.006171
3648	1.2e-2648	0.0784	0.004395	0.006668	0.006302	0.004036
3598	2.3e-2633	0.109	0.003405	0.005691	0.005323	0.003044
3548	4.7e-2622	0.1178	0.001869	0.004156	0.003787	0.00151
3498	3.2e-2620	0.1273	-0.0008	0.001454	0.001089	-0.00116
3448	1.2e-2617	0.1379	-0.00074	0.001519	0.001153	-0.00109
3398	7.1e-2618	0.1541	-0.00167	0.000571	0.000206	-0.00202

146
143
141
14
139
138
137
137
137
137

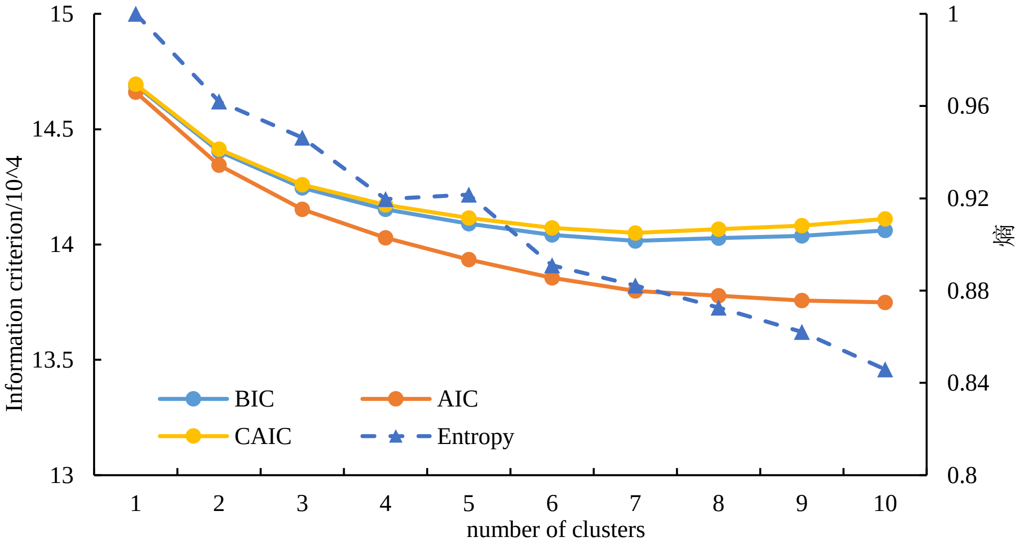

Supplement: S1 Dataset — (PDF) [file pone.0301293.s001.pdf]
