# Supplementary material for: Analysis of factors influencing the degree of accidental injury of bicycle riders considering data heterogeneity and imbalance
Source: PLoS One. 2024 May 14;19(5):e0301293. doi: 10.1371/journal.pone.0301293 (PMC11093317; doi:10.1371/journal.pone.0301293)
Supplement: S1 Table — (PDF) [file pone.0301293.s002.pdf]

Table 2. Distribution of featured variables describing cluster characteristics.

**Variables**		**C1 (59.59)**	**C2 (29.46)**	**C3 (10.95)**
**Time**				
Season	Spring	21.4	24.38	25.2
	Summer	32.24	34.6	31.09
	Autumn	27.88	25.76	27.96
	Winter	18.48	15.25	15.75
Day of Week	Working day	72.6	72.64	76.56
	Weekend	27.4	27.36	23.44
Hour of Crash	Morning Peak	15.66	10.58	13.41
	Evening Peak	25.02	28.54	29.51
	Off-Peak	59.33	60.88	57.08
**Drivers**				
Gender	Male	57.36	62.73	56.95
	Female	42.64	37.27	43.05
Age	18～25 years old	15.06	16.99	11.62
	26～40 years old	26.75	33.14	30.2
	41～65 years old	42.99	38.23	45.36
	＞65 years old	15.2	11.64	12.82
Failure to yield	Yes	9.31	2.41	12.7
	No	90.69	97.59	87.3
View was blocked	Yes	7.68	6.8	8.64
	No	92.32	93.2	91.36
Inattention	Yes	6.82	8.41	7.99
	No	93.18	91.59	92.01
**Cyclists**				
Gender	Male	80.26	85.2	77.94
	Female	19.74	14.8	22.06
Age	＜18 years old	19.1	20.35	19.76
	18～25 years old	19.26	14.08	19.29
	26～40 years old	24.21	25.55	24.58

Variables		C1 (59.59)	C2 (29.46)	C3 (10.95)
	41～65 years old	30.55	35.05	31.86
	＞65 years old	6.88	4.97	4.52
Initial Direction of Travel	Forward	48.67	70.13	25.2
	Reverse	44.23	12.36	68.82
	Downward	7.09	17.5	5.98
Failure to yield	Yes	26.79	27.31	6.99
	No	73.21	72.69	93.01
Violation of Traffic Control Device	Yes	12.51	2.98	0.32
	No	87.49	97.02	99.68
Inattention	Yes	3.42	6.95	3.23
	No	96.58	93.05	96.77
**Vehicle**				
Pre-Event Movement	Going Straight	32.94	69.03	13
	Turn left	21.75	12.09	10.68
	Turn right	38.64	3.99	57.98
	Start/Stop	5.82	2.24	12.74
	Other	0.86	12.66	5.6
Vehicle Type	Passenger Cars	53.79	55.57	57.65
	Multi-purpose vehicle	22.49	19.87	21.81
	Trucks / Buses	23.72	24.56	20.54
Manner of Collision	Front-to-Front	66.49	50.68	61.96
	Left Sideswipe	12.53	14.64	8.7
	Right Sideswipe	20.97	34.69	29.34
Age of vehicle	0～5 years	41.85	39.18	43.17
	6～10 years	24.75	23.33	24.57
	11～15 years	21.36	21.92	22.89
	＞15 years	12.03	15.58	9.36
**Roadway**				
Roadway Surface Condition	Non-Trafficway or Driveway Access	0.25	0.99	73.57

Variables		C1 (59.59)	C2 (29.46)	C3 (10.95)
	Dry	91.58	90.7	25.94
	Wet	8.17	8.31	0.5
Relation to Junction	Intersection	91.93	20.8	3.72
	Near the intersection	7.66	12.51	6.94
	Non-intersection	0.41	66.69	89.34
Road Type	Travel Lane	44.74	86.41	13.6
	Bicycle Lane/Paved Shoulder/Parking Lane	4.25	10.11	2.69
	Sidewalk/Cross Walk/Driveway Access	51.02	3.48	83.71
**Environmental**				
Weather	Clear	82.58	82.27	85.62
	Cloudy	11.72	11.97	10.92
	Rain/Snow and other bad weather	5.7	5.75	3.46
Light Condition	Daylight	77.5	70.37	89.96
	Dark-Not Lighted	1.92	7.18	1.2
	Dark-Lighted	16.02	17.54	4.92
	Dawn/Dusk	4.56	4.91	3.91
Traffic Control Device	None	13.87	92.78	81.19
	Traffic signals	54.73	6.2	1.1
	Stop/Yield signs	31.4	1.03	17.71

Note: the units of the figures in the table are "%".
