# Supplementary material for: Analysis of factors influencing the degree of accidental injury of bicycle riders considering data heterogeneity and imbalance
Source: PLoS One. 2024 May 14;19(5):e0301293. doi: 10.1371/journal.pone.0301293 (PMC11093317; doi:10.1371/journal.pone.0301293)
Supplement: S2 Table — (PDF) [file pone.0301293.s003.pdf]

Table 3. Evaluation index of different BN models in the C1 accident cluster

| Models | Index | | | | |
|---|---|---|---|---|---|
| | *Accuracy* | *Sensitivity* | *Specificity* | *G-mean* | *AUC* |
| C1_HC | 0.907 | 1.000 | 0.000 | 0.000 | 0.575 |
| C1_TPDA | 0.907 | 1.000 | 0.000 | 0.000 | 0.575 |
| C1_ROS_HC | 0.892 | 0.841 | 0.943 | 0.891 | 0.960 |
| C1_ROS_TPDA | 0.848 | 0.758 | 0.939 | 0.843 | 0.926 |
| C1_SMOTE_HC | 0.776 | 0.763 | 0.789 | 0.776 | 0.858 |
| C1_SMOTE_TPDA | 0.828 | 0.824 | 0.832 | 0.828 | 0.907 |
| C1_ADASYN_HC | 0.779 | 0.763 | 0.794 | 0.779 | 0.854 |
| C1_ADASYN_TPDA | 0.829 | 0.828 | 0.830 | 0.829 | 0.911 |

Table 4. Evaluation index of different BN models in the C2 accident cluster

| Models | Index | | | | |
|---|---|---|---|---|---|
| | *Accuracy* | *Sensitivity* | *Specificity* | *G-mean* | *AUC* |
| C2_HC | 0.802 | 1.000 | 0.000 | 0.000 | 0.577 |
| C2_TPDA | 0.801 | 0.999 | 0.000 | 0.000 | 0.645 |
| C2_ROS_HC | 0.708 | 0.641 | 0.776 | 0.705 | 0.769 |
| C2_ROS_TPDA | 0.678 | 0.599 | 0.757 | 0.673 | 0.755 |
| C2_SMOTE_HC | 0.713 | 0.679 | 0.746 | 0.712 | 0.782 |
| C2_SMOTE_TPDA | 0.731 | 0.707 | 0.756 | 0.731 | 0.811 |
| C2_ADASYN_HC | 0.704 | 0.677 | 0.730 | 0.703 | 0.777 |
| C2_ADASYN_TPDA | 0.706 | 0.680 | 0.733 | 0.706 | 0.774 |

Table 5. Evaluation index of different BN models in the C3 accident cluster

| Models | Index | | | | |
|---|---|---|---|---|---|
| | *Accuracy* | *Sensitivity* | *Specificity* | *G-mean* | *AUC* |
| C3_HC | 0.937 | 1.000 | 0.000 | 0.000 | 0.425 |
| C3_TPDA | 0.937 | 1.000 | 0.000 | 0.000 | 0.435 |
| C3_ROS_HC | 0.963 | 0.925 | 1.000 | 0.962 | 0.995 |
| C3_ROS_TPDA | 0.916 | 0.848 | 0.985 | 0.914 | 0.972 |
| C3_SMOTE_HC | 0.930 | 0.910 | 0.951 | 0.930 | 0.973 |
| C3_SMOTE_TPDA | 0.943 | 0.923 | 0.964 | 0.943 | 0.983 |
| C3_ADASYN_HC | 0.898 | 0.889 | 0.907 | 0.898 | 0.955 |
| C3_ADASYN_HC | 0.918 | 0.887 | 0.950 | 0.918 | 0.970 |

Table 6. Evaluation index of different BN models in the OD accident cluster

| Models | Index | | | | |
|---|---|---|---|---|---|
| | *Accuracy* | *Sensitivity* | *Specificity* | *G-mean* | *AUC* |
| OD_HC | 0.879 | 1.000 | 0.000 | 0.000 | 0.615 |
| OD_TPDA | 0.879 | 1.000 | 0.000 | 0.000 | 0.617 |
| OD_ROS_HC | 0.681 | 0.627 | 0.735 | 0.679 | 0.741 |
| OD_ROS_TPDA | 0.685 | 0.638 | 0.732 | 0.683 | 0.757 |
| OD_SMOTE_HC | 0.678 | 0.660 | 0.697 | 0.678 | 0.740 |
| OD_SMOTE_TPDA | 0.743 | 0.730 | 0.755 | 0.742 | 0.817 |
| OD_ADASYN_HC | 0.701 | 0.655 | 0.746 | 0.699 | 0.757 |

| | | | | | |
|---|---|---|---|---|---|
| OD_ADASYN_TPDA | 0.702 | 0.662 | 0.741 | 0.700 | 0.774 |
